# RF Heating Effects in CEST NMR with Hyperpolarized ^129^Xe Considering Different Spin Exchange Kinetics and Saturation Schemes

**DOI:** 10.1002/cphc.202401037

**Published:** 2025-02-05

**Authors:** David Hernandez‐Solarte, Leif Schröder

**Affiliations:** ^1^ Division of Translational Molecular Imaging Deutsches Krebsforschungszentrum (DKFZ), Im Neuenheimer Feld 280 69120 Heidelberg Germany; ^2^ Graduate Research Center 2260 BIOphysical Quantitative Imaging Towards Clinical Diagnosis (BIOQIC) Department of Radiology Charité – Universitätsmedizin Berlin, CCM, Charitéplatz 1 10117 Berlin Germany; ^3^ Department of Physics and Astronomy Ruprecht-Karls University Heidelberg Im Neuenheimer Feld 226 69120 Heidelberg Germany; ^4^ German Consortium for Translational Cancer Research (DKTK) Core Site Heidelberg Im Neuenheimer Feld 280 69120 Heidelberg Germany

**Keywords:** NMR, CEST, Hyperpolarized xenon, RF heating

## Abstract

Chemical exchange saturation transfer (CEST) improves the sensitivity of NMR but depending on the spin exchange kinetics, it can require substantial RF energy deposition to label magnetization. Potential side effects like RF‐induced heating may occur and must be monitored. Here, we explore the parameter space considering not only undesired heating but also efficient CEST build‐up (depolarization rate), spectral resolution (line width), and subsequent effects like changes in chemical shifts of CEST responses. We present a systematic study to compare conventional block pulse with shaped‐pulse saturation and quantify how the effective average saturation power impacts these parameters. Pulse shape and nominal excitation bandwidth, however, turned out to have little impact on acquired z‐spectra and temperature changes. This study illustrates how different exchange kinetics define different regimes of suitable RF power within the dynamic range of fully saturable magnetization from hyperpolarized ^129^Xe. Temperature‐related changes in the resonance frequency of bound spins were also quantified for the two Xe hosts CB6 and CrA‐ma and put into context for typically used CEST acquisition parameters, including the stability of the magnetic field.

## Graphical abstract

Chemical exchange saturation transfer (CEST) with different spin exchange kinetics can require substantial RF energy deposition to label magnetization in separate chemical environments. The parameter space with optimum saturation should be judged not only concerning unwanted heating but also consider efficient CEST build‐up (depolarization rate), spectral resolution (line width) and subsequent effects like changes in chemical shifts of CEST responses.

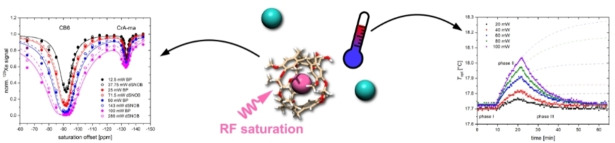

## Introduction

Temperature is an important parameter in many samples and organisms that are investigated by nuclear magnetic resonance (NMR). This includes dynamic molecular processes studied in analytical spectroscopy, as well as biomedical applications where non‐invasive temperature monitoring can be desired during imaging diagnostics or for certain hyperthermia treatments. Spectroscopy and imaging (MRI) measurements can both be the cause of temperature changes (through RF‐induced heating), but they also offer the feature to sense temperature *in situ*. This is typically done through quantifying small changes in the Larmor frequency, as expressed by the chemical shift δ of the detected nuclei, given in ppm. Based on this concept, MR thermometry can be performed in different approaches.[^1^, ^2^] A prominent example in clinical context is the proton resonance frequency (PRF) method^[3]^ due to the abundance of the water proton signal in many samples. As an alternative, ^129^Xe thermometry[^4^, ^5^, ^6^] is motivated by the higher sensitivity ∂δ/∂T compared to the PRF method. Furthermore, spin exchange rates in systems with chemical exchange are obviously temperature‐dependent and cause changes in line width and observable saturation transfer efficiency. RF‐induced heating is of particular interest for the emerging field of chemical exchange saturation transfer (CEST) applications, where the double role of MR as a) the origin of energy deposition and b) a sensing modality is under‐investigated and motivates further studies.

This is especially true when CEST and ^129^Xe NMR are combined in the HyperCEST method^[7]^ to further boost the signal amplification of CEST by observing exchanging hyperpolarized spins of the dissolved hyperpolarized noble gas. Various host systems have been studied for this method^[8]^ and a complex parameter space must be considered when including temperature changes in the system description: The resonance frequency of transiently bound Xe as well as its exchange rate between pools A and B (*k*_BA_) depend both on the sample temperature. *k*_BA_, on the other hand, determines the required RF power to achieve efficient saturation transfer. Faster exchange, which is made accessible by the large chemical shift range of ^129^Xe or by ParaCEST agents,^[9]^ requires higher *B*_1_ amplitudes to unleash the full potential of saturation transfer. Importantly, HyperCEST agents have been shown to cover a large range of spin turnover rates (the combination of the exchange rate and the host occupancy). Lack of sufficient RF power for faster exchange therefore yields poorer outcome than for agents providing slow exchange.^[10]^ Applications using more efficient agents are also more prone to temperature changes throughout the experiment and the need for updating on‐resonant saturation conditions should be investigated.

The HyperCEST concept for ultra‐sensitive MRI has been the focus of some recent translational studies regarding *in vivo* applications of a (yet unfunctionalized) Xe host.[^11^, ^12^, ^13^] These studies relied on CB6, a macrocyclic host that provides rather variable exchange conditions for reversible binding of ^129^Xe, depending on the solvent conditions: in pure water, the signal of bound Xe is practically undetectable in conventional spectra due to relatively fast exchange,^[10]^ whereas the presence of cations or interaction partners at the portals can significantly reduce the exchange rate.^[14]^ Excessive RF power, however, causes only broadening of the CEST response^[15]^ and should be avoided for unwanted heating with no contrast benefits. One aim of this study is therefore to also investigate the interplay of exchange kinetics, suitable saturation power, and the concomitant effects of RF‐induced heating. The search for optimum saturation schemes with a high CEST effect but SAR‐conform (SAR: specific absorption rate) RF power deposition for *in vivo* applications has become an important aspect in this context. The comparison of CEST performance from different agents, with different saturation schemes, and at different field strengths *B*_0_ requires a meaningful absolute measure for RF power with regard to the potential heating effects. A previous study^[16]^ parameterized different shaped RF pulse schemes according to the nominal flip angle. This prevents useful comparison of saturation schemes. We will demonstrate that the flip angle is a misleading measure in CEST experiments and that both cw and shaped‐pulse saturation can be compared in a meaningful way when simply the RF power in Watt is used to assess heating effects.

The range of applied saturation pulse amplitudes is broad, and literature on the relevance of RF heating in this context is limited. While it is expected in many cases that the effects are barely detectable, other situations may arise where RF heating does indeed become relevant. Some CEST protocols use relatively high saturation power (>20 μT over several hundreds of ms or even seconds) due to exchange conditions that require a strong *B*_1_ amplitude to act on spins with a short residence time in the pool which is to be saturated. Examples can be found both in ^1^H CEST[^9^, ^17^] and ^19^F CEST.^[18]^ We will elaborate in this study that even though such *B*_1_ values correspond to transmit powers of less than 1 W in microimaging setups, such saturation can cause measurable effects because the sample size is on the order of 1 g of water. A scaling for larger samples should apply accordingly.

Regarding small samples, RF‐induced heating could also be relevant for other CEST applications like in structural biology,[^19^, ^20^, ^21^] where unknown temperature changes can affect the interpretation of kinetic parameters and chemical shift values. Conventional sample temperature control and monitoring usually happens through an external gas stream (variable temperature unit, VTU) that does not report directly on the conditions inside the sample. Deviations from the target temperature might cause inaccuracies that warrant further investigations for (Hyper)CEST studies.

Investigating RF‐induced heating with ^129^Xe is not only motivated by using this nucleus in HyperCEST studies. Following proper calibration, the NMR signals can be used for NMR thermometry. Both the signal of free dissolved ^129^Xe and reversibly trapped atoms change with different temperature sensitivity.[^4^, ^22^] The signal of Xe trapped in cryptophane‐A (CrA) is more sensitive to temperature changes, but it typically comes with lower signal intensity. This study therefore includes data on how different types of ^129^Xe NMR signals are suitable to follow temperature changes *in situ*. In the case of Xe@cryptophane‐A (CrA), the chemical shift separation between bound and free Xe has been described to decrease linearly with 0.29 ppm/K for *T*>298 K with increasing temperature,^[22]^ even though the chemical shift of dissolved Xe in water follows an overall parabolic behavior. This latter, non‐linear effect has been attributed to density changes of the surrounding solvent as a critical aspect. Moreover, different solvents also yield different sensitivity.^[23]^ Both aspects motivate to study the two most commonly used HyperCEST agents (CrA and curcurbit[6]uril, CB6) in more detail and with an absolute temperature reference instead of the indirect VTU approach.

Taking all these aspects together, this study comprises a detailed analysis of heating effects, including the observable chemical shift and amplitude of CEST response of dissolved, hyperpolarized ^129^Xe for two types of hosts with different exchange kinetics and under different saturation schemes. The CEST concept serves both as an application case for RF‐induced heat deposition of various degrees and as an analytical tool for the otherwise inaccessible signal from CB6. Results are co‐validated with an optical temperature sensor *in situ*. We further discuss the supposed benefits of shaped pulses (dSNOB saturation^[24]^) reported in previous studies^[16]^ where a comparison with conventional cw saturation (block pulse) had not been done in a fair and systematic approach. This new work includes suitable scaling of pulse amplitudes using the area under the curve $\int_{0}^{t}B_{1}\left(t\right)dt$ when comparing the effects of RF power deposition.

## Methods and Materials

### Saturation Transfer Efficiency Considerations

Considering high saturation power, *P*_sat_, and thus pondering on accepting RF heating side effects is particularly relevant for CEST sites with relatively high exchange rates *k*_BA_ from the saturated into the detected pool. ^129^Xe with its large chemical shift range provides favorable conditions in as much as it enables selective saturation even for *k*_BA_>10^3^ s^−1^ without significant spillover effect. It turns certain Xe hosts into powerful HyperCEST agents. *P*_sat_ should be sufficient to delete the magnetization during the residence time 1/*k*_BA_. This is considered by introducing the labeling efficiency^[25]^ α (see below). The comparison of saturation conditions should always be done within the sensitive part of the CEST dynamic range, *i. e*., by avoiding conditions close to full saturation. A previous study^[16]^ claimed to optimize the performance of a HyperCEST agent via pulse sequence development but compared z‐spectra under almost meaningless conditions with practically full saturation. The interplay of host concentration, exchange rate, and applied *P*_sat_ is therefore important for choosing the experimental parameters. For Hyper‐CEST, the responses in z‐spectra can be described by an exponential Lorentzian;^[26]^ hence, the negative peak shape becomes highly compressed when approaching full saturation. We have already pointed out in previous work^[27]^ that saturation responses can only be approximated by a linear behavior for saturations <20—30 %. Any responses stronger than this should only be compared by considering the predicted line shape from the full HyperCEST (FHC) solution.

Regarding strong responses, CB6 is a prominent example of a host where the CEST build‐up benefits from high *B*_1_. The exchange rate for Xe with CB6 depends on the presence of counter‐ions at the carbonyl group‐bearing portals. Reported values are high and range between *k*_BA_=2100 s^−1^ in pure water^[10]^ and ca. 900…1000 s^−1^ in PBS.^[28]^ In fact, the effective release rate of saturated Xe from the host can be decomposed into a dissociative and a degenerate exchange contribution. The latter one only contributes the term *k*[Xe]_eq_, with [Xe]_eq_ being the concentration of unbound Xe in chemical equilibrium and *k*≈120 M^−1^ ms^−1^.^[28]^ The concentration of dissolved Xe in HyperCEST studies is usually in the high μM to low mM regime (the Xe solubility in PBS is *s*=3.97 mM/bar).^[28]^ The degenerate exchange contribution is therefore on the order 10^−1^ ms^−1^ or less, while the dissociative contribution dominates with 1.1 ms^−1^ and yields the above‐mentioned *k*_BA_. This number is relevant for the maximum on‐resonant depolarization rate, $\lambda_{\mathrm{depol}}$ ,^[15]^ as the essential parameter that determines the depth of the CEST response. It depends on *B*_1_ and therefore on *P*_sat_:

(1) $$\lambda_{\mathrm{depol}}\left(B_{1}\right)\approx f_{B}k_{BA}\frac{{\left(\gamma B_{1}\right)}^{2}}{{\left(\gamma B_{1}\right)}^{2}+k_{\mathrm{BA}}^{2}}\equiv f_{B}k_{BA}\alpha$$

where $f_{B}=\left[Xe_{\mathrm{host}}\right]/{\left[\mathrm{Xe}\right]}_{\mathrm{eq}}$ is the fraction of bound Xe *vs*. free Xe in chemical equilibrium.

The ratio ${\left(\gamma B_{1}\right)}^{2}/k_{\mathrm{BA}}^{2}\equiv \epsilon^{2}$ is of central importance. Herein, the gyromagnetic ratio has to be used as $\gamma =-2\pi \times 11.77\times 10^{6}\;{\left(\mathrm{Ts}\right)}^{-1}$ . It should be noted that $B_{1}\propto \sqrt{P_{\mathrm{sat}}}$ and therefore any change in *P*_sat_ proportionally translates into a change in ϵ . Equation (1) has been derived for cw saturation, but qualitatively similar arguments hold for ongoing pulsed saturation with negligible inter‐pulse delays.

Developing a meaningful experimental design warrants taking first a closer look at actual numbers for CB6 HyperCEST experiments to decide how increased *P*_sat_ contributes to improved CEST responses despite unavoidable RF heating. With exchange rates being on the order *k*_BA_ ≈10^3^ s^−1^, this is usually the dominant term in ϵ . Even a strong saturation with *B*_1_=33.3 μT as used in the original CB6 imaging study^[10]^ yields ${\left(\gamma B_{1}\right)}^{2}={\left(2463\;s^{-1}\right)}^{2}$ and is indeed comparable to $k_{BA}^{2}$ in pure water, but not dominant. We see from Equation (1) that for ${\left(\gamma B_{1}\right)}^{2}\approx k_{\mathrm{BA}}^{2}$ , α is only ca. 0.5, and it was shown later that the depolarization rate for CB6 in pure water increases for *B*_1_ up to ~100 μT.^[15]^ We therefore have to assume a poor labeling efficiency in general for this host. Nevertheless, *B*_1_=33.3 μT (corresponding to *P*_sat_=115 mW; based on a calibration that a 90° reference pulse lasts 38 μs at *P*=32 W, translating to *B*_1_=555.62 μT on our system) is nowadays considered excessive saturation. This seemingly contradictory rating can be explained once more with the above‐mentioned line shape: the often‐used combinations of applied saturation time and CB6 concentrations in the high nM to low μM range for *in vitro* experiments produce already rather strong saturation effects for *P*_sat_<100 mW. Higher power would build up a similar response even faster, but the visible result is mainly a broadening in the shoulder of the response. *In vivo* studies on clinical scanners anyway cannot apply such high *B*_1_ due to SAR limitations. Pulses in this present study will therefore not exceed values comparable to a block pulse with *P*_sat_=100 mW (or *B*_1_=31 μT, ${\left(\gamma B_{1}\right)}^{2}={\left(2294\;s^{-1}\right)}^{2}$ ). The previous optimization study for CB6^[16]^ used *B*_1_=9 μT for the stronger block pulses, hence ${\left(\gamma B_{1}\right)}^{2}={\left(666\;s^{-1}\right)}^{2}$ , which is even for PBS solutions of CB6 dominated by the exchange term, and we assume ϵ<1 in typical experiments for CB6.

**Figure 1:**
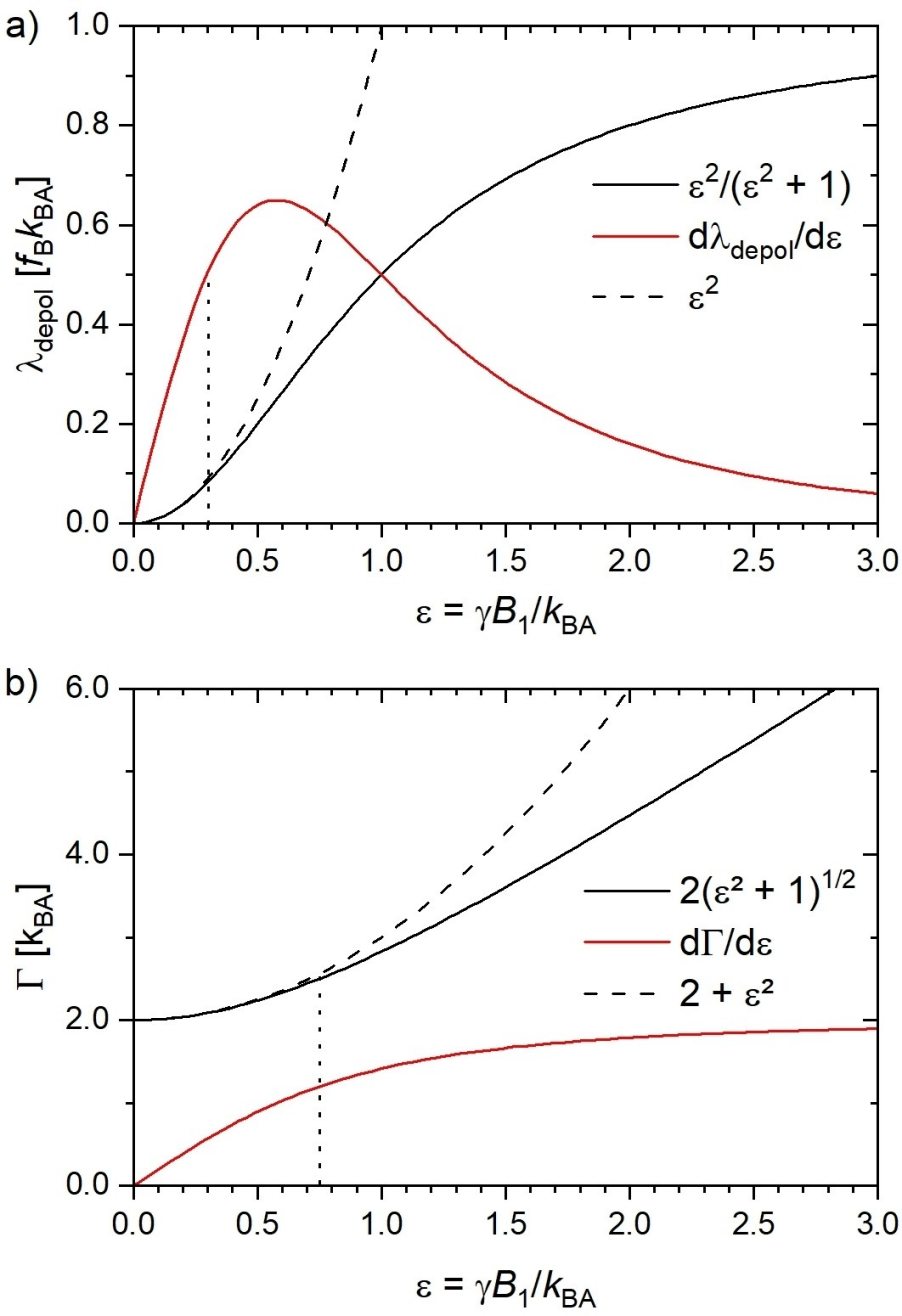
(**a**) Impact of the scaling parameter ϵ from $\gamma B_{1}=\epsilon k_{\mathrm{BA}}$ on the depolarization rate $\lambda_{\mathrm{depol}}$ . A quadratic approximation is only valid for ϵ<0.3 . (**b**) Impact of ϵ on the CEST line width width Γ . Most conditions for CB6 fulfill ϵ<0.75 where the function can be approximated as a quadratic behavior $\Gamma \approx k_{\mathrm{BA}}\left(2+\epsilon^{2}\right)\propto P_{\mathrm{sat}}$ .

Using the definition of ϵ , the depolarization rate reads $\lambda_{\mathrm{depol}}\left(\epsilon \right)=f_{B}k_{\mathrm{BA}}\frac{\epsilon^{2}}{1+\epsilon^{2}}$ . This is plotted in Figure 1(a) together with $\frac{d\lambda_{\mathrm{depol}}}{d\epsilon }=2f_{B}k_{\mathrm{BA}}\frac{\epsilon }{{\left(\epsilon^{2}+1\right)}^{2}}$ . We see that $\lambda_{\mathrm{depol}}\left(\epsilon \right)$ can be approximated by a quadratic behavior $f_{B}k_{\mathrm{BA}}\epsilon^{2}$ for ϵ<0.3 , but not further. For testing CB6 (and particularly CrA with a slower exchange rate) under different saturation conditions, a wider range of ϵ must be considered. Using $\epsilon =\gamma B_{1}/k_{\mathrm{BA}}=c\sqrt{P}/k_{\mathrm{BA}}=\kappa \sqrt{P}$ (*c* given by the pulse calibration, κ being defined as a summarized scaling factor), the experimentally observed depolarization rate should follow the function.

(2) $$\lambda_{\mathrm{depol}}\left(P_{\mathrm{sat}}\right)=f_{B}k_{\mathrm{BA}}\frac{\kappa^{2}P_{\mathrm{sat}}}{1+\kappa^{2}P_{\mathrm{sat}}}.$$

This will be tested experimentally by acquiring z‐spectra with different *P*_sat_.

### Exchange Kinetics and Line Shape Considerations

Another important aspect to consider is the impact of increased *P*_sat_ on line broadening. Excessive saturation should be avoided not only since it causes more heating but also unwanted line broadening without increasing the depth of the CEST response.^[15]^ The line width for on‐resonant saturation of the host pool is given by the FHC description^[15]^ as:

(3) $$\Gamma \left(B_{1}\right)\approx 2\sqrt{{\left(\gamma B_{1}\right)}^{2}+k_{BA}^{2}}.$$

Despite working in the low‐α regime, a qualitative estimation regarding the width Γ and possible off‐resonant saturation affecting the baseline in the z‐spectrum are useful to predict expected changes related to adjusting *P*_sat_ within typically used ranges. Similar to α , Γ is also dominated by the exchange rate for CB6. We therefore do not expect the nominal bandwidth of the applied RF pulse to be relevant for the observed Γ . The dependence on *B*_1_, however, gives important insights. Using again $\gamma B_{1}=\epsilon k_{\mathrm{BA}}$ , the line width reads $\Gamma =2k_{\mathrm{BA}}\sqrt{\epsilon^{2}+1}$ . This is plotted in Figure 1(b) together with the derivative $\frac{d\Gamma }{d\epsilon }\;=2k_{\;\mathrm{BA}}\frac{\epsilon }{\sqrt{\epsilon^{2}\;+1}}$ and we can see that $\Gamma \left(\epsilon \right)$ can be approximated by the quadratic function $\Gamma \approx k_{\mathrm{BA}}\left(2+\epsilon^{2}\right)$ for ϵ<0.75 . A block pulse with *P*_sat_=50 mW on our system would yield ϵ≈0.77 for exchange conditions in pure water. We therefore expect the saturation responses for low to medium RF power to broaden quadratically with *B*_1_, hence linearly with *P*_sat_. For values beyond 50 mW, the increase is almost linear with *B*_1_ (always assuming no full saturation where the line shape would be dominated by the shoulder of the exponential Lorentzian).

When approaching almost full signal depletion for on‐resonant saturation, it is questionable whether higher *P*_sat_ is any beneficial. Grynko et al. mentioned off‐resonant effects that affect the baseline between the CEST response and direct saturation of the free pool.^[16]^ Such a dropping baseline may eventually impact the resolution of the two peaks. Although these effects are indirectly included in the description for Γ , they can be understood from the Bloch‐McConnell equations. These equations exist in different notations,^[29]^ but the matrix notation by Woessner^[30]^ provides useful insights. $\gamma B_{1}=\omega_{1}$ is again an important parameter and must be compared to the saturation offset Δω from the Larmor frequency of the respective pool. In the rotating frame of reference, the ratio $\omega_{1}/\Delta \omega$ is important to define the orientation of the effective field ${\vec{B}}_{\mathrm{eff}}$ about which the magnetization $\vec{M}\left(t\right)$ precesses. For strong pulses with $\gamma {\vec{B}}_{1}=\left(\gamma B_{1},0,0\right)$ or small offsets corresponding to $\Delta \vec{\omega }=\left(0,0,\Delta \omega \right)$ , the effective field is aligned with ${\vec{B}}_{1}$ such that $\vec{M}$ only rotates in the *y‐z*‐plane. As Δω increases, ${\vec{B}}_{\mathrm{eff}}$ tilts towards the z‐axis. For $\Delta \omega >\gamma B_{1}$ , $\vec{M}$ precesses on a cone that does not touch the *x‐y*‐plane anymore. This precession geometry for large offsets keeps the *M_y_* component rather limited and preserves most of *M_z_*. But the corresponding cross terms in the matrix become more relevant for off‐resonant saturation when *B*_1_ increases (and ${\vec{B}}_{\mathrm{eff}}$ tilts towards the *x*‐axis): the terms $dM_{z}/dt\propto -\omega_{1}M_{y}$ and $dM_{y}/dt\propto +\omega_{1}M_{z}$ become more important, while at the same time the terms $dM_{x}/dt\propto +\Delta \omega M_{y}$ and $dM_{y}/dt\propto -\Delta \omega M_{x}$ are not directly affected by the increase in *B*_1_. Overall, the loss in *M*_z_ magnetization becomes stronger and the baseline in the z‐spectrum drops. This should only be accepted if the CEST response itself keeps growing.

### Production and Delivery of Hyperpolarized ^129^Xe

Spin polarization of ^129^Xe was achieved with a gas mix of 2‐vol.% of Xe (^129^Xe at natural abundance of 26.4 %) combined with 10 % N_2_ and 88 % He. This mix underwent spin‐exchange optical pumping (SEOP) in a custom‐designed continuous‐flow polarizer equipped with a laser emitting at 795 nm with 150 W (emission bandwidth ~0.5 nm; BrightLock, QPC Lasers, Sylmar, CA). The achieved ^129^Xe polarization degree of ~20 % corresponds to ~10^4^‐fold NMR signal enhancement. The hyperpolarized Xe gas was continuously transferred through 0.25” PFA tubing into the NMR magnet bore and dispersed into the sample via fused silica glass capillaries (inner/outer diameter of 250/350 μm, respectively; Polymicro Technologies/Molex; part#: 1068150030) at a flow rate of 100 mL/min at intervals of 15 s. This was followed by a waiting time of 3 s to allow the remaining bubbles to collapse. The above‐mentioned solubility of Xe in water in combination with a total gas mix pressure of 4.5 bar yields a Xe concentration in solution of ca. 395 μM at 2 % Xe fraction.

### Sample Preparation

The two Xe hosts CrA‐ma and CB6 were chosen for their different exchange kinetics, with CB6 providing a ca. 100‐fold more efficient gas turnover rate in pure water than CrA‐ma.^[10]^ Both hosts were dissolved in DI water to obtain stock solutions of 10 μM for CrA‐ma and CB6. These were diluted to 8 μM (CrA‐ma) and 2 μM (CB6; reduced concentration to prevent from excessive CEST responses due to the faster exchange) unless otherwise noted. pH was adjusted for improved solubility in Tris buffer to 9.

### Xe NMR Spectroscopy

All studies were done on a 400 MHz wide‐bore NMR spectrometer (*B*_0_=9.4 T; Bruker Biospin, Ettlingen, Germany; software: ParaVision 6.0 with Topspin 3.1 for ParaVision). Excitation and signal detection were achieved with a 10 mm double‐resonant ^129^Xe/^1^H transmit/receive probe head (^1^H channel used for shimming and comparisons of RF heating at 400 MHz). All ^129^Xe z‐spectra were acquired from free Xe in solution, serving as the bulk pool (referenced to 0 ppm). The 90° reference pulse was 38 μs at *P*=32 W (*B*_1_=555.62 μT) and converts into a 90° block pulse of 1 ms duration with a standard reference (sr) power *P*_sr_=46.18 mW. This parameter is useful when comparing saturation pulses for setups with larger samples that would also require higher *P*_sr_.

Previous studies motivated the dSNOB pulse, which has been suggested to provide a more efficient approach.^[24]^ The conversion factor of a shaped pulse relative to a reference block pulse can be obtained from the spectrometer software Topspin 3.1 to ensure comparable absolute energy deposition. This conversion factor is 2.86 for the dSNOB pulse. The following two types of pulse sequences were used (graphical illustration is given in the Suporting Information, Figures. S1/S2):

*Thermometry*: The off‐resonant application of RF irradiation with subsequent detection of the dissolved Xe signal was implemented for this sequence with a loop of 128 iterations where each one consisted of a preparation with xenon bubbling delivery into the sample and collapsing time (as explained before), followed by a 10‐second RF (pulsed) saturation, and a readout pulse of 1063 μs with a Gaussian profile (2 kHz bandwidth). The sequence was designed to get a baseline value of the chemical shift where for the first 16 loops the saturation power was set to zero, followed by 24 loops of actual RF application in which the saturation power and duty cycle were kept constant for these iterations. In the remaining 88 loops, the power was again set to zero to let the sample recover to ambient (VTU‐controlled) temperature. Each iteration has a duration of 30 seconds, for a total duration of 64 minutes per experiment. This sequence was performed with the 110 MHz channel and the 400 MHz channel as well (data shown in Figure S5), where the only difference is that the channel was switched before and after the 10‐second saturation time; hence the readout was always acquired with the Xe channel. The saturation was performed using dSNOB and block pulses with variable amplitude and referenced to block pulses with *P*_sat_=20 100 mW, i. e. ca. 43 % … 217 % of *P*_sr_.

*Z‐spectroscopy*: The saturation preparation was followed by a global readout with a 38 μs block pulse (equivalent to the 1063 μs Gaussian profile used before) on the frequency of free dissolved ^129^Xe. The FID signal was acquired for up to 4 s (204‘078 data points) with a bandwidth of 25.510 kHz/230 ppm in a pseudo‐2D sequence. The saturation offset in ppm was incremented in every iteration where free dissolved Xe served as the bulk pool (referenced to 0 ppm). The direct saturation response was sampled±10 ppm around the solution pool, and the CEST response from both types of Xe hosts from −75 to −115 ppm for CB6, and from −120 to −145 ppm for CrA‐ma. An additional three acquisitions were used at the beginning as dummy scans with saturation offset at +200 ppm to achieve a steady‐state Xe magnetization in the solution. The saturation duration from this sequence ranged from 2 seconds up to 20 seconds, with power settings ranging from *B*_1_=11 μT (block pulse; *P*_sat_=12.5 mW=27 % *P*_sr_) to 52 μT (dSNOB peak power; *P*_sat_=286 mW=619 % *P*_sr_). Further conversions for pulse parameters are given in the supplementary tables S1/S2.

### Reference Thermometry

**Figure 2:**
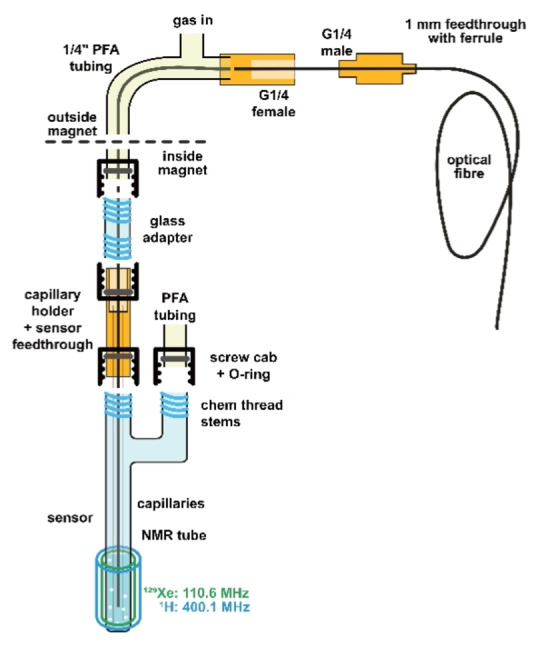
Experimental setup with inserted optical temperature sensor. Xe gas dispersion occurs in a pressure‐tight volume at 4.5 bar abs.

As a reference temperature sensor, we used a Micronor ASY‐0673 A, a 3 m long nonmagnetic optical probe with a current convertor within the measurement range of 0–80 °C with an accuracy of ±0.2 °C. The data is recorded using the Micronor USB software TPMeter 10 FTC‐DIN‐GT‐HT with an acquisition repetition that matches the NMR pulse sequence repetition time. The probe was placed inside the NMR tube as shown in Figure 2, 3 using a feed‐through that maintains the gas pressure inside the sample.

**Figure 3:**
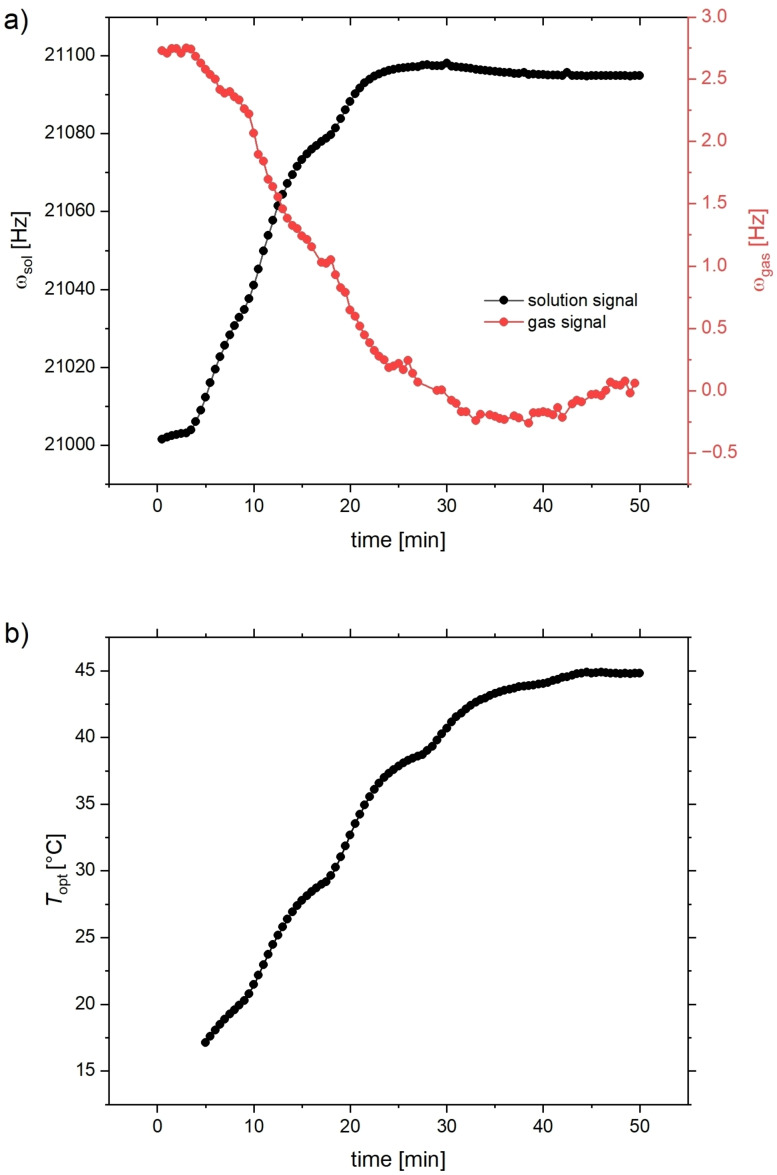
Heating calibration experiment. (**a**) Larmor frequencies of gas and solution peak in Hz as a function of time. For 20 min<*t*<30 min, the gas still shows mainly linear behavior, whereas changes in the solution peak already slow down. Dents in both curves correlate and reflect variability in the VTU performance (confirmed in b). (**b**) Recording for *T*_opt_ from the optical sensor.

The VTU system of the spectrometer was operated at 400 SLM gas flow and was used to perform a temperature calibration of the chemical shift $\delta_{\mathrm{sol}}$ of dissolved ^129^Xe to obtain the function $\delta_{\mathrm{sol}}\left(T\right)$ . The calibration curve was measured using the optical sensor reading instead of the value measured by the VTU since the optical sensor is in direct contact with the sample and Xe gas interaction.

### Data Post‐Processing

Spectra were obtained from the pseudo‐2D data sets that comprise a stack of 1D spectra. The signal intensity of the free solution signal at 0 ppm was integrated with Topspin (projection of the sum of columns). This data was imported into OriginPro^®^ using the NMR Plugin (oNMR 8). The nonlinear fitting toolbox was used to obtain the peak position from spectra with 5 Hz line broadening previously that was previously applied in Topspin.

## Results and Conclusions

### Calibrations and Field Drift Corrections

A quantification of temperature changes from the chemical shift of ^129^Xe NMR signals in aqueous solution should be carefully calibrated. Prior knowledge for precise correction is required because the observed chemical shift is a superposition of temperature‐related effects and of the intrinsic drift of the magnetic field *B*_0_ since ^129^Xe detection is not necessarily assisted by a ^2^H lock channel (particularly not on clinical MRI systems). For clinical scanners, the drift for the water resonance can be ~20 Hz/hr on a 3 T system^[31]^ or ~40 Hz/hr on a 7 T system.^[32]^ This has not been considered in previous HyperCEST studies.

However, evaluating drift‐related changes in the observed chemical shift of dissolved Xe over time, *i. e*., $\delta_{\mathrm{sol}}\left(t\right)$ , is time‐consuming on a well‐adjusted spectrometer since even without a lock channel, the (constant) drift compensation reduces the observable effects quite significantly. The remaining drift in Hz/hr is small enough that its evaluation can be influenced by the phasing of the ^129^Xe signal when determining its exact line position. Nevertheless, the drift itself can be eliminated as unwanted uncertainty when working with frequency separations instead of absolute chemical shifts. Since the signal of dissolved Xe is usually referenced to the signal of Xe in gas phase at $\delta_{\mathrm{gas}}=$ 0 ppm, evaluation of both signals (that are equally affected by the drift) would in principle provide a way to obtain $\delta_{\mathrm{sol}}$ as a function of temperature that is not skewed by the magnet drift. A drift‐independent calibration of $\delta_{\mathrm{sol}}\left(T\right)$ would allow deriving $T\left(\delta_{\mathrm{sol}}\right)$ in subsequent measurements, if (and only if) both signals are acquired. This requirement holds even for an almost perfectly compensated drift because the gas reference itself might also depend on temperature, even though $\partial \delta_{\mathrm{gas}}/\partial T<\partial \delta_{\mathrm{sol}}/\partial T$ can be assumed from experimental observations. In fact, previous work^[33]^ has referenced all values of 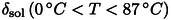 to a “fixed” Larmor frequency of ^129^Xe gas at room temperature and 1 atm.

We decided to perform an initial set of experiments with a temperature sweep and broadband detection to obtain first insights into the relative magnitudes of the observed frequency changes. These spectra were acquired with 250 ppm bandwidth, with the center frequency adjusted such that both signals of dissolved Xe at $\delta_{\mathrm{sol}}\approx 190$ ppm and of the gas at $\delta_{\mathrm{gas}}\approx 0\;\;ppm$ from the headspace on top of the 1 mL liquid sample were detected.

It is important to note that the type of gas delivery used here, *i. e*., interval bubbling, also has an impact on the sample temperature. It was expected that the gas coming from the SEOP setup may not have completely cooled down to room temperature during the transit time into the NMR detection volume (the pumping cell is operated at *T*_SEOP_>120 °C). However, we observed that the overall effect of bubble dispersion causes a net cooling effect that is registered in real‐time by the optical temperature sensor *in situ*.

The system was therfore allowed to equilibrate for ca. 10 min under the repetitive bubbling delivery conditions (15 s bubbling, 3 s waiting time) and the equilibrium temperature *T*_1_ was registered. The Larmor frequencies $\omega_{\mathrm{gas}/\mathrm{sol}}$ for both resonances were determined (from the two fitted signals after careful phasing) over a time course of ca. 50 min. After a few minutes, the nominal VTU temperature was changed from 15 °C to 50 °C and *T*_opt_ was registered until a stabilized value *T*_opt,max_ could be read. The frequency changes within this time frame are plotted in Figure 3(a). This plot reflects the temperature‐related changes as well as the drift and the relative magnitude of $\partial \omega_{\mathrm{gas}}/\partial T$ in comparison to the drift and to $\partial \omega_{\mathrm{sol}}/\partial T$ . The correlated changes in *T*_opt_ observed by the optical sensor are plotted for comparison in Figure 3(b). A comparison of both frequency plots illustrates that the gas signal shows a monotonous upfield move while *T* increases. For *t*>40 min, the gas signal only shows the impact of the magnet drift. For orientation, it should be noted that the isolated magnet drift obtained from fitting a linear behavior to the data of $\omega_{\mathrm{gas}}\left(t>40\;\;\min\right)$ was ca. 0.030 Hz/min=1.8 Hz/hr (0.016 ppm/hr). The full temperature change of 35 °C caused a larger observable frequency change of ca. 3 Hz for the gas peak. This number is underestimated, as it was partially compensated by the drift. On the other hand, the solution peak frequency changes by almost 100 Hz throughout the experiment and reaches a maximum at *t*≈27 min, i. e. *T*≈40 °C. The large *T*‐related effects make it more challenging to quantify the drift from this data.

**Figure 4:**
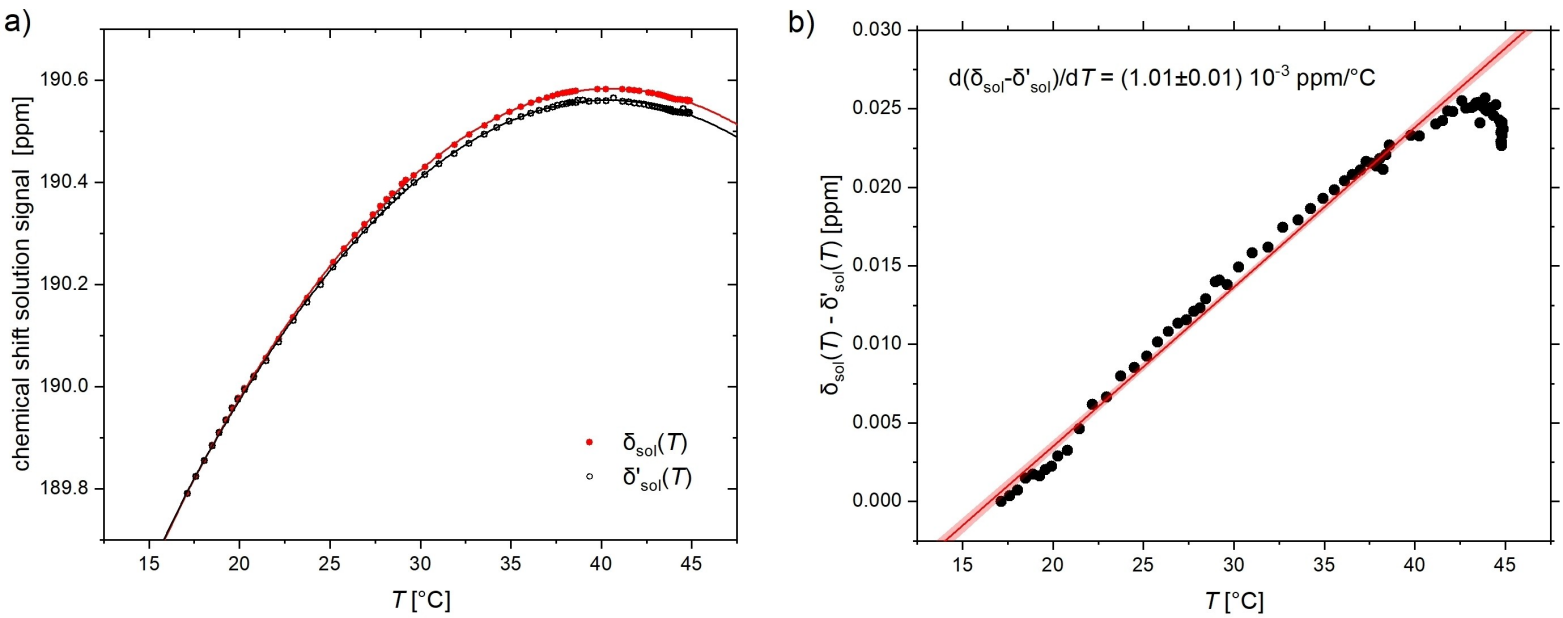
Chemical shift separation between the gas phase signal (upfield) and the dissolved ^129^Xe signal (downfield) while sweeping the sample temperature (heating 15 °C→50 °C; data for cooling 50 °C→15 °C available in the Suporting Information). (**a**) The data follows a parabolic behavior and illustrates that the VTU set temperature of 50 °C is not reached inside the sample. The full symbols represent $\delta_{\mathrm{sol}}\left(T\right)$ with data obtained including the impact of *T* on the reference $\delta_{\mathrm{gas}}$ , the open symbols show $\delta_{\mathrm{sol}}^{'}\left(T\right)$ with data obtained from using a fixed reference with $\partial \delta_{\mathrm{gas}}/\partial T=0$ and no magnet drift correction. (**b**) Difference between the two evaluations, illustrating a systematic deviation only for high temperatures. The linear fit represents the change of the gas signal with temperature.

Similar data were also collected for a cooling experiment with switching the VTU set point back from 50 °C to 15 °C (data shown in the Suporting Information Figure S4). Both the heating and the cooling data sets were used to determine the chemical shift separation $\delta_{\mathrm{sol}}\left(T\right)$ of the solution peak from the gas peak for each time point and retrospectively assign it to the corresponding value of *T*_opt_ *(t)*. Results for the heating experiment are plotted in Figure 4(a). The observed behavior $\delta_{\mathrm{sol}}\left(T\right)$ was fitted to a parabolic function that was also predicted in theoretical descriptions,^[33]^ even though the latter work used a fixed $\partial \delta_{\mathrm{gas}}/\partial T=0$ . This function $\delta_{\mathrm{sol}}\left(T\right)$ can be used as an absolute reference for different experimental sessions and spectrometers that record both the solution and the gas phase signal.

In another evaluation, now with a referencing approach similar to that used in Ref.^[33]^ the gas peak frequency was determined only once (at the beginning) and then used as a constant reference throughout the temperature sweep. Even though this approach does not eliminate the magnet drift, this curve $\delta_{\mathrm{sol}}^{'}\left(T\right)$ was compared to the other parabola $\delta_{\mathrm{sol}}\left(T\right)$ to rank the relative uncertainties. It could already be seen in Figure 3(a) that the magnet drift must be small compared to $\partial \delta_{\mathrm{gas}}/\partial T$ . Hence, the two parabolas in Figure 4(a) differ systematically at high temperatures, where the deviation from using a presumably temperature‐independent $\delta_{\mathrm{gas}}$ (in the data with open symbols) has become more obvious. This linear trend (see Figure 4(b)) actually represents an estimate of the temperature‐dependence of the gas signal and could be quantified as $\partial \delta_{\mathrm{gas}}/\partial T$ =(1.01±0.01)×10^−3^ ppm/°C. If the magnet drift over 40–60 min were the dominant error, this would show up most prominently for last recorded data points, *i. e*., systematically for high temperatures in the heating experiment and low temperatures in the cooling experiment. But this pattern could not be observed. Moreover, we can extrapolate from the drift estimated in Figure 3(a) that this type of error does not exceed ca. 1.5 Hz (0.013 ppm) over the 50‐min course of the experiment.

Some aspects should be mentioned regarding this approach for calibrating the parabolic temperature dependence. The independent absolute temperature measurement inside the sample through the optical sensor, *T*_opt_, allows a quick calibration of $\delta_{\mathrm{sol}}\left(T\right)$ when temperature cycling of the sample is done. This provides advantages with little consequences for the overall accuracy:

– Thanks to the prompt response of the optical sensor, all data can be recorded as dynamic *T*‐sweep and this approach is faster because it does not require reaching a steady state for each temperature.
– The fastest temperature change is at the beginning; the data is therefore sparser at low *T* for heating and becomes denser at high *T*. For the cooling approach, this is *vice versa*. The lowest accuracy is given for the beginning of the heating experiment with low data density in the steep regime of the parabola; in this regime, the temperature changes by 0.9 °C between two data points.
– However, data from both sweeping directions practically overlap, and no systematic deviation could be observed. This illustrates that even in the range of fast sweeping, reproducible results are obtained.

### Consistency Check for Magnet Drift Estimation During RF Heating

Both parabolas in Figure 4(a) may be considered to quantify temperature changes from the observed solution peak in CEST experiments with RF presaturation. However, the typical CEST experiment only records a narrow spectral range around the solution pool signal that serves as the chemical shift reference for the applied saturation offsets. The gas peak, however, is usually omitted, even if a gas phase were present in the detection volume. The drift in Hz/min is rather small along the course of an experiment if the total acquisition time does not exceed 1 h. Hence, this inaccuracy can be kept under control. If $\delta_{\mathrm{gas}}$ is not available, then the observed changes in the solution peak frequency should be referenced to the calibration curve $\delta_{\mathrm{sol}}^{'}\left(T\right)$ because $\delta_{\mathrm{sol}}\left(T\right)$ would yield an underestimated change in *T* due to the ignored contribution of $\partial \delta_{\mathrm{gas}}/\partial T$ .

The next set of experiments focused on the evaluation of only $\delta_{\mathrm{sol}}$ at high spectral resolution. The signal of dissolved Xe was acquired under different temperature stimuli to serve two purposes: first, it provides an orientation regarding the magnitudes of temperature changes that occur during RF heating with typical *P*_sat_ and recovery along the course of the experiment. Second, we tested a consistent identification of the magnet drift in a retrospective approach.

**Figure 5:**
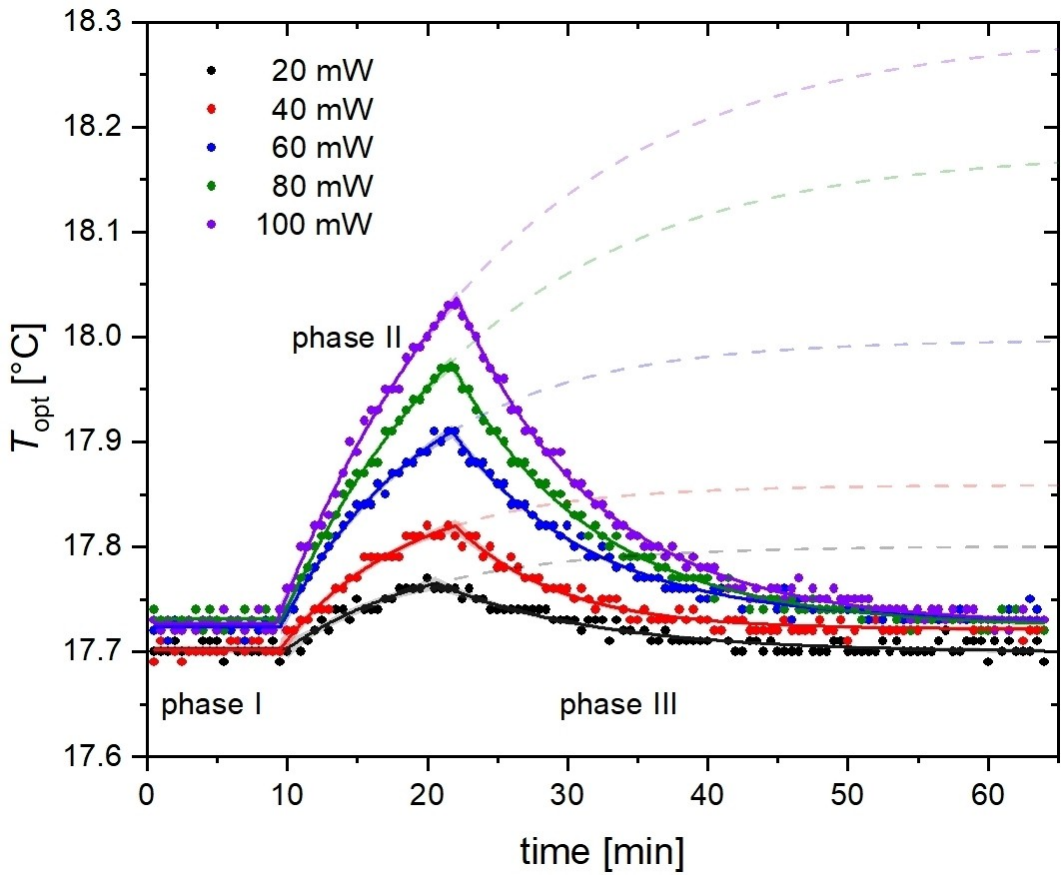
RF heating effects registered by the optical sensor after application of dSNOB pulses with variable power (100 Hz nominal bandwidth) with variable power for *t*_sat_=10 s. *P*_sat_ is given as the block pulse equivalent. A piecewise fit according to Eq. 4 is performed. The exponential increase is extended beyond phase II (dashed lines) to illustrate the eventually reachable temperature *T*_eq_.

Again, an accurate quantification of the drift requires knowledge of the true sample temperature. Any unintended fluctuations might be easily the dominating contribution to changes in the resonance frequency over the time course of a typical experiment. We followed the RF heating effects for different RF pulse powers with the optical temperature sensor and through the signal of dissolved Xe via $\delta_{\mathrm{sol}}$ . To this end, we acquired data sets with 16 dummy scans (power of the saturation pulse set to zero) at the beginning, followed by 24 iterations with pulsed irradiation for 10 s (*P*_sat_=20…100 mW block pulse equivalent), and finally 88 dummy scans for monitoring the cool‐down. Figure 5 clearly demonstrates that the sample initially maintains a steady temperature, *T*_1_, with no further cooling from bubbling once the actual data recording starts for phase I with negligible power deposition.

**Figure 6:**
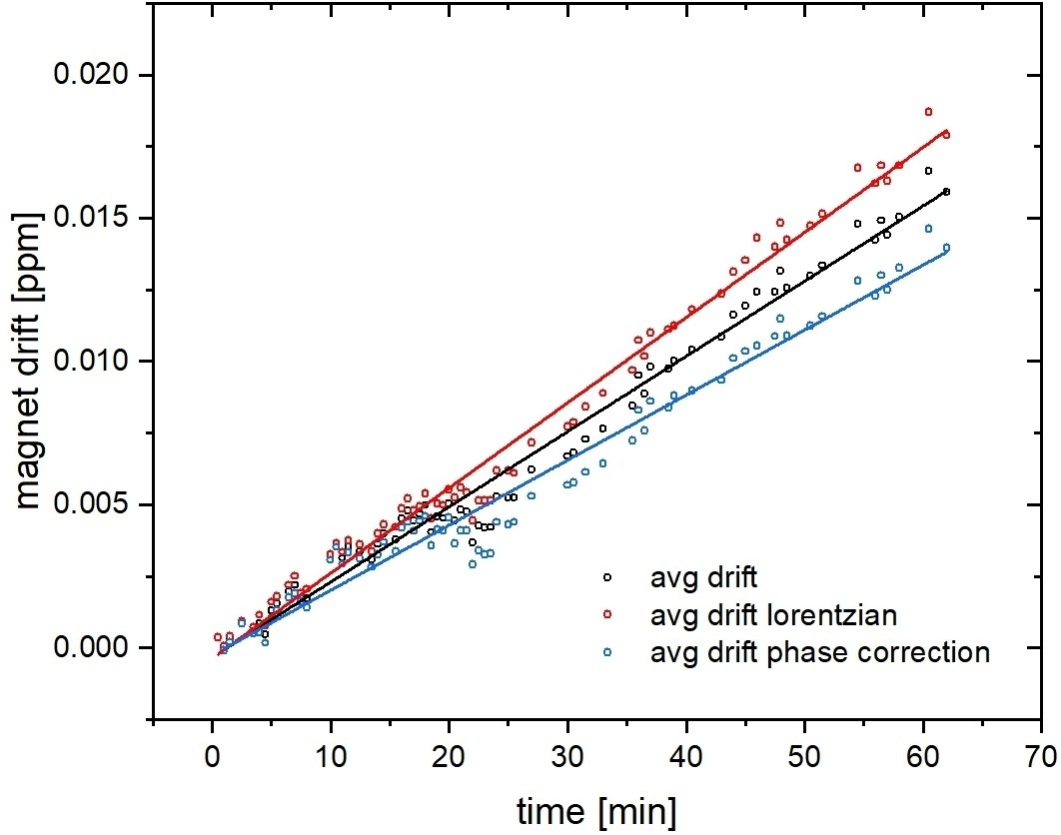
Magnet drift quantification after using an inverse approach based on the calibration of $\delta_{\mathrm{sol}}^{'}\left(T\right)$ . The values represent the average deviation of temperature‐related changes in chemical shifts for all five data sets in Figure 5 from the observed chemical shifts over the course of the 60‐min experiment.

Before comparing the impact of different RF power, we first performed the above‐mentioned independent quantification of the magnet drift based on the initially acquired calibration curve $\delta_{\mathrm{sol}}^{'}\left(T\right)$ . To this end, we decompose the observed change in chemical shift into temperature‐related effects and the intrinsic magnet drift: Changes in *T*_opt_ *(t)* from the starting temperature *T*_1_=~17.7 °C in Figure 5 are first converted with the slope around *T*_1_ in $\delta_{\mathrm{sol}}^{'}\left(T_{1}\right)$ back into changes in chemical shifts. These values are then subtracted from the observed chemical shifts $\delta_{\mathrm{sol}}\left(T,t\right)$ and yield a linear deviation over time as shown in Figure 6. For these drift calculations taken from all five datasets in Figure 5, two options were used to evaluate the observed peak position for $\delta_{\mathrm{sol}}$ : a) determining the position of the maximum in magnitude spectra, and b) evaluating phased real spectra by fitting a Lorentzian line shape. The fitted Lorentzian line shapes yield a consistently larger drift of 2.97(4)×10^−4^ ppm/min=1.97(3) Hz/h. The magnitude spectra yield a drift of 2.27(5)×10^−4^ ppm/min=1.51(3) Hz/h and the average of both methods 2.63(5)×10^−4^ ppm/min=1.75(3) Hz/h. This is in excellent agreement with the drift derived from the gas peak for *t*>40 min in Figure 3(a).

### Quantitative Comparison of Heating Effects

A more detailed quantitative analysis of the RF heating recorded in Figure 5 was done by fitting the data to a piece‐wise function for phases I–III:

(4) $$T\left(t\right)=\left\{\begin{array}{cc} T_{1} & \mathrm{for}\;t\;<t_{1}\\ T_{1}+\Delta_{\max}\left(1-e^{-\left(t-t_{1}\right)/\tau_{1}}\right) & \mathrm{for}\;t_{1}\leq t<t_{2}\\ \Delta_{h}\left(e^{-\left(t-t_{2}\right)/\tau_{2}}\right)+T_{\mathrm{final}} & \mathrm{for}\;t_{2}\;<t \end{array}\right.$$

where *t*_1_ and *t*_2_ are the time points where effective heating starts and ends, respectively. During RF‐induced heating in phase II, *T* increases exponentially with time constant τ_1_ towards a new equilibrium, *T*_eq_=*T*_1_+Δ_max_. For time reasons, the heating is stopped at *t*_2_ before this equilibrium is reached. Phase III is governed by exponential cooling with τ_2_ towards the final temperature *T*_final_ (assuming accurate performance of the VTU, *T*_1_=*T*_final_ should apply). The observed temperature increase is given by $\Delta_{h}\left(t_{2}\right)=\left(T_{\mathrm{eq}}-T_{1}\right)\left(1-e^{-\left(t_{2}-t_{1}\right)/\tau_{1}}\right)$ and the maximum achievable heating by $\Delta_{\max}=\left(T_{\mathrm{eq}}-T_{1}\right)$ . Table 1 summarizes the fitting results for comparing the impact of increased power regarding $\Delta_{h}\left(t_{2}\right)$ , $\Delta_{\max}$ , and the time constants. In general, the data can be modeled with high confidence using the piecewise fit; the 95 % confidence level bands barely exceed the width of the plotted line.

**Table 1:** Fitting results for observed heating parameters with increasing RF power analyzed from Figure 5. Fit parameter results±SD are given.

*P*_sat_ [mW]	*T*_1_ [°C]	Δ_h_(*t*_2_) [°C]	*Δ*_max_ [°C]	*τ*_1_ [min]	*τ*_2_ [min]
20	17.7026±0.0015	0.0628±0.0029	0.098±0.037	10.50±6.43	11.42±1.26
40	17.7022±0.0015	0.1179±0.0029	0.157±0.019	9.01±2.05	7.31±0.50
60	17.7242±0.0014	0.1859±0.0029	0.273±0.029	10.75±1.92	9.90±0.37
80	17.7316±0.0014	0.2438±0.0028	0.446±0.056	15.29±2.80	10.18±0.27
100	17.7267±0.0016	0.3106±0.0030	0.566±0.057	16.24±2.41	10.04±0.25

**Figure 7:**
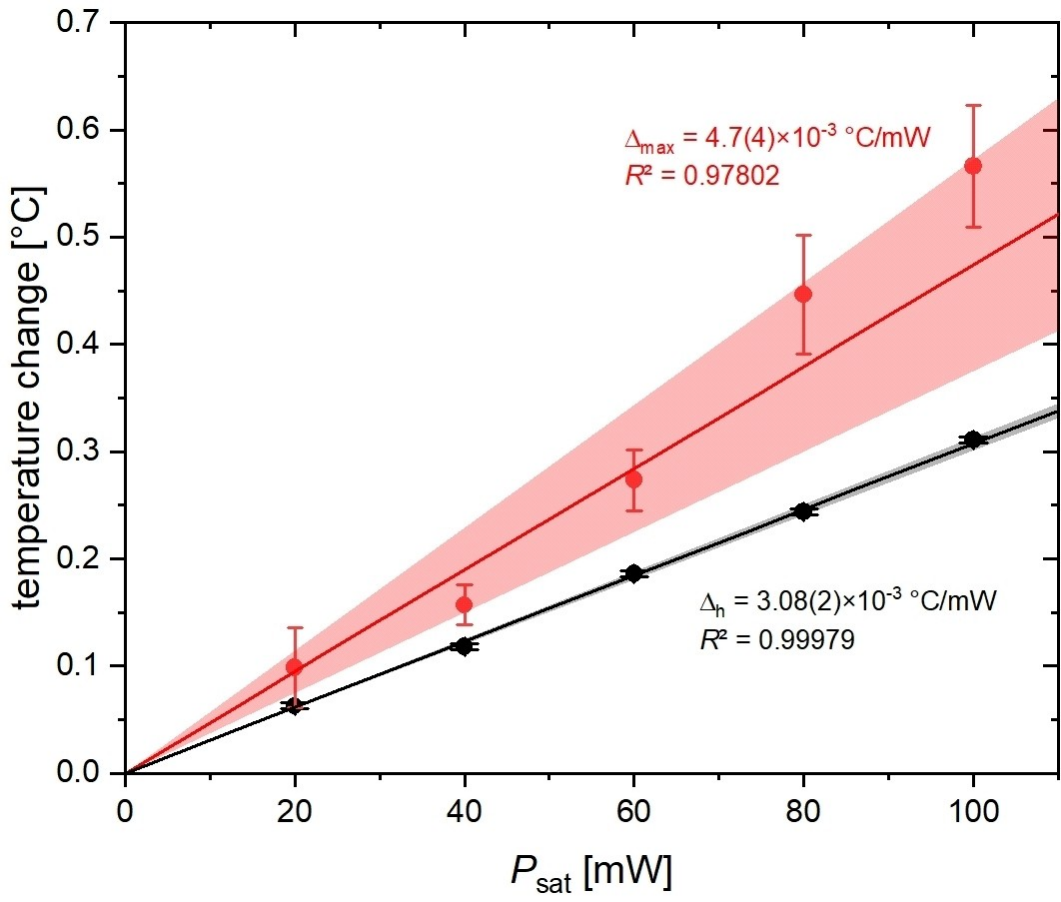
Achieved heating effects $\Delta_{h}$ and maximum temperature change $\Delta_{\max}$ from plots in Figure 5 for 1 mL of water as a function of applied RF power, *P*_sat_. Numbers are given for fitting a linear behaviour (including the SD in the last significant digit) and 95 % confidence level bands are shown.

The maximum observed heating effect at time point *t*_2_, $\Delta_{h}\left(t_{2}\right)$ , clearly depends on *P*_sat_, as seen in Figure 7. $\Delta_{h}\left(P_{\mathrm{sat}}\right)$ follows extremely accurately the expected linear behavior. The values for $\Delta_{\max}\left(P_{\mathrm{sat}}\right)$ come with larger errors due to the limited duration of phase II. Nevertheless, they can also be modeled with a linear behavior. All maximum heating effects remained within 0.6 °C. The time constants, however, do not follow a clear trend but illustrate that an equilibrium is reached after ca. 50–60 min. Heating, which is governed by the chosen RF power and the heat capacity of the water sample, occurs with a time constant comparable or longer to that of cooling in phase III. The latter is influenced by the heat dissipation through the surrounding gas stream of the VTU system (in fact, the VTU sensor does not really sense that *T* inside the sample changes) and is specific to the experimental setup used in this study.

Taken together, the comparison of pulses with different saturation strength is best done based on their power given in W. The pulse shape is irrelevant for the total heating effect, only *t*_sat_ determines the new *T*_eq_, as shown in the Suporting Information Figure S6. Previous work^[16]^ used the flip angle for long selective pulses (*t*_pulse_⩾30 ms) with values exceeding 1000°. This parameter becomes meaningless, especially for very efficient CEST agents like CB6: any tilted magnetization with a transverse component immediately experiences strong dephasing due to Larmor frequency jumps between the two exchanging pools. Hence, large flip angles cannot really be achieved. A flip angle of 1200° represents more than three full revolutions, occurring over 30 ms. During this time frame, Xe spins jump multiple times between the free and the bound state in CB6. The two Larmor frequencies differ by at least 75 ppm (or more, depending on the solvent), which is already at *B*_0_=3 T a separation of ca. 2.6 kHz. This means that transverse Xe magnetization is in anti‐phase orientation only 190 μs after entering the CB6 cavity. In fact, CB6 has been described as one of the most potent *T*_2,ex_ agents despite the lack of a paramagnetic center.^[34]^ This rapid loss of phase coherence compared to the pulse duration makes the definition of a defined flip angle a meaningless parameter in this context and requires the use of other parameters to predict the expected effects (see also next section).

### Implications for CEST Spectroscopy with Different Exchange Kinetics and Suitable Parameter Choice.

**Figure 8:**
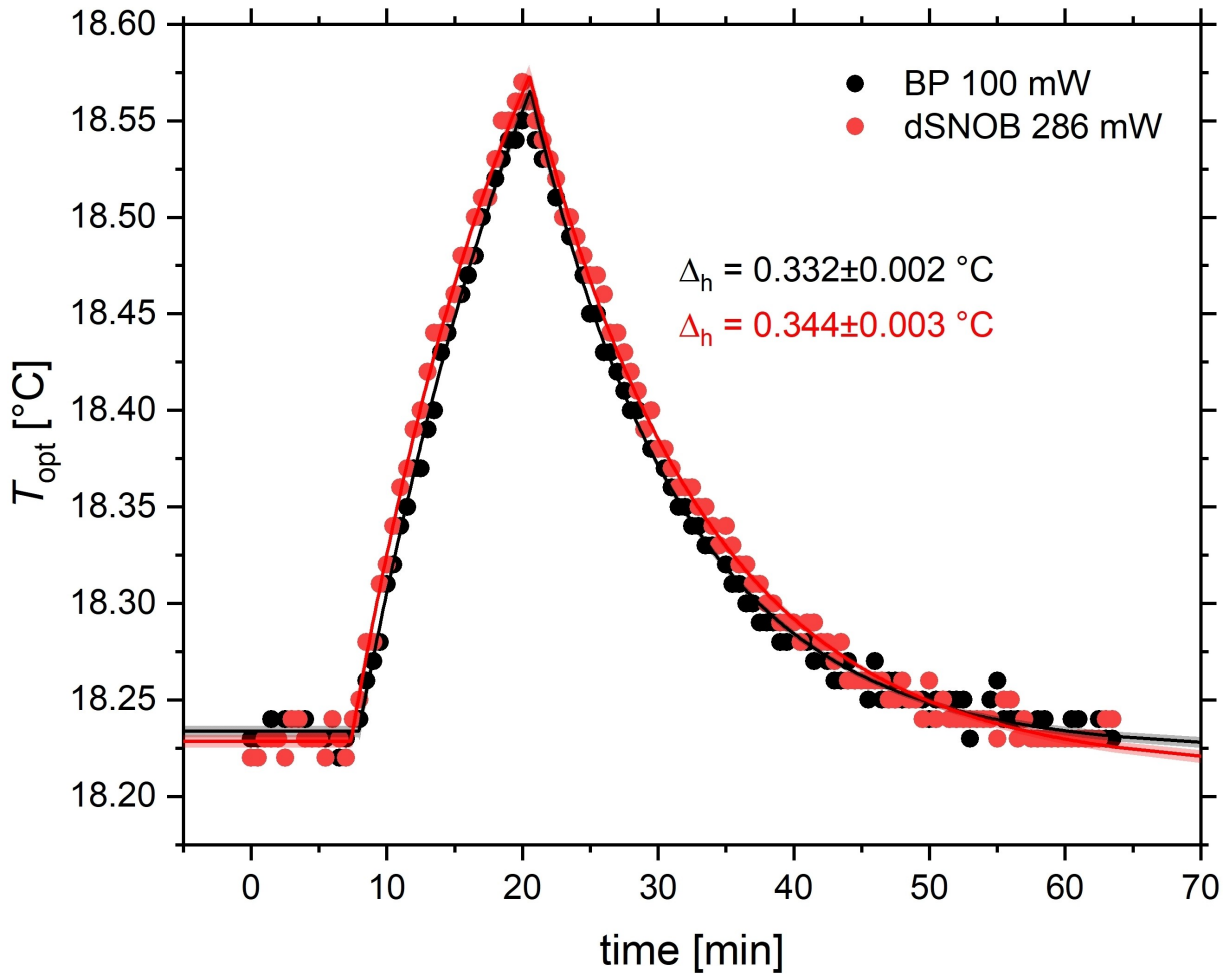
Heating effect from a 100 mW block pulse and a 286 mW dSNOB (100 Hz bandwidth) pulse power as registered by the optical sensor. Results are consistent with Figure 5 and both pulses cause a heating effect of $\Delta_{h}\approx$ 0.338 °C with negligible difference (less than the accuracy of the optical sensor).

For CEST experiments, we decided to compare the two Xe hosts CrA‐ma (8 μM) CB6 (2 μM). Their CEST responses are separated by ca. 40 ppm. The differences in exchange kinetics motivate the investigation of different saturation pulse schemes. The heating effect induced by the different pulse shapes is expected to be identical if the proper scaling factor is applied. For validation, we performed two exemplary heating experiments using each pulse with the strongest cw power equivalent and monitored the sample conditions with the optical temperature sensor. Figure 8 illustrates that the observed changes $\Delta_{h}\approx$ 0.338 °C are practically identical in phase II. Note that for data in Figure 8, the 100 mW saturation is not applied as a long cw pulse but as a series of block pulses that match the length of the dSNOB pulse (*t*_pulse_=11.44 ms duration, 100 Hz bandwidth) to achieve the same saturation time (*t*_sat_=10 s) as in Figure 5. However, we did not observe any differences when the block pulse scheme was applied as a single, 10‐s cw pulse (data not shown). The number of pulses is irrelevant, as is the bandwidth/duration of the dSNOB pulse, as long as the total pulse train length *t*_sat_ remains unchanged.

**Figure 9:**
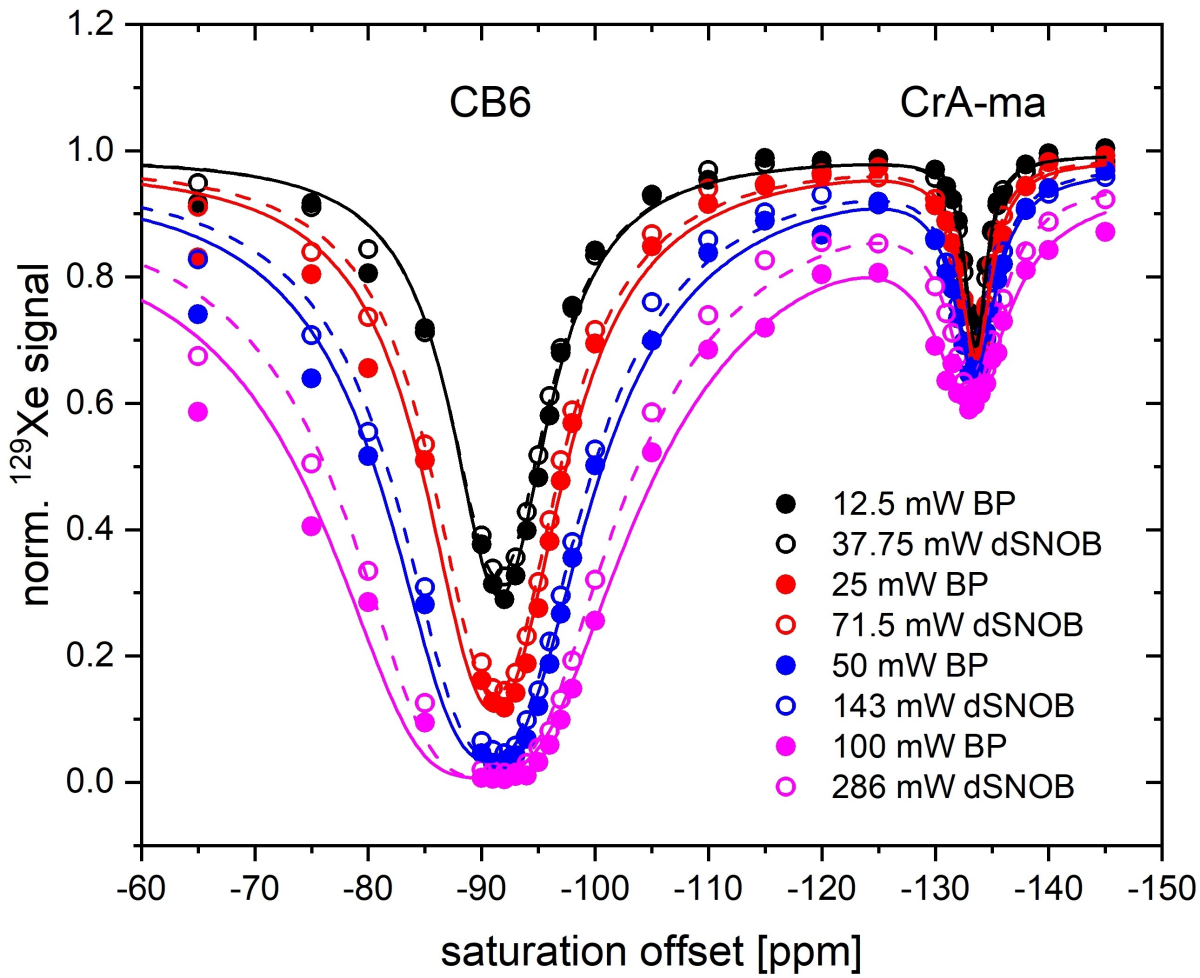
HyperCEST responses of CB6 (2 μM) and CrA‐ma (8 μM) for block pulse and dSNOB saturation (25 Hz nominal bandwidth) at different saturation powers. Only the CB6 signal benefits from stronger saturation.

We then compared the achievable CEST responses for both saturation schemes throughout a range of different *P*_sat_. Similar work had been done in a previous study^[16]^ without taking proper scaling factors into account. The pulse power for the block pulse ranged from *P*_sat_=12.5 mW (10.98 μT) to 100 mW (31.06 μT), and the peak power of dSNOB pulses (25 Hz nominal bandwidth, duration *t*_pulse_=45.76 ms) accordingly from 35.75 mW to 286 mW. A full z‐spectrum with 50 saturation offsets (*t*_sat_=5 s) as shown in Figure 9 was recorded in ~21 min. Sufficiently dense sampling is necessary to evaluate the shape of the CEST response without loss of information. Sparse sampling as done in a previous work for hypsec pulses^[16]^ would not allow analyzing the line shapes with sufficient accuracy. As the power increases, the response of CrA‐ma primarily changes in width rather than maximum CEST effect. The faster exchange of Xe with the CB6 cavity definitely benefits from higher *P*_sat_ to show stronger CEST responses. However, it also exhibits broadening of the saturation profile, as expected from the theory described in the Methods section. At this concentration of CB6, no further increase of *t*_sat_ beyond 5 s or *P*_sat_>100 mW is beneficial.

**Figure 10:**
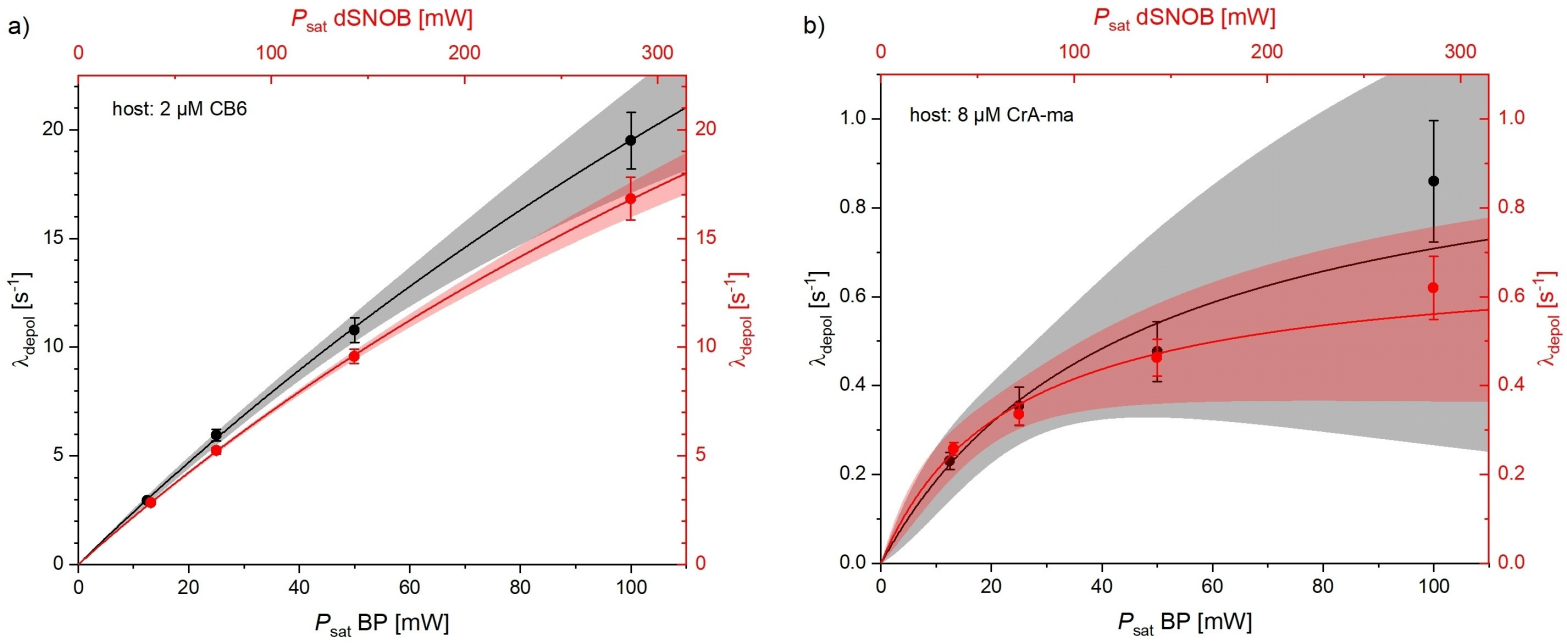
Increase in depolarization rate with *P*_sat_ from z‐spectra in Figure 9 for saturation with block pulses (black) in comparison to dSNOB pulses (red). Data is fitted to Eq. 2 and shown with 95 % confidence level bands. (**a**) The HyperCEST response for the faster exchanging host CB6 follows perfectly the predicted behavior in the low‐α regime. (**b**) For CrA‐ma with much slower exchange, the uncertainty is much larger, as the signal does not change a lot with increasing *P*_sat_.

A quantitative analysis of the impact of increasing *P*_sat_ is shown in Figure 10 where $\lambda_{\mathrm{depol}}$ as obtained from fitting the spectra in Figure 9 is analyzed. In accordance with previous studies regarding the FHC description,^[35]^ the conditions with changes in the low‐α regime (as for CB6 in this case) are much more informative than those where minor changes happen around stronger saturation (as for CrA‐ma). Figure 10 illustrates how the saturation response grows with *P*_sat_ according to Eq. 2 with high confidence in the regime below the maximum depolarization rate $f_{B}k_{\mathrm{BA}}\approx 80$ (average of the two fits) for [CB6]=2 μM. The minor changes for the CrA‐ma response are linked to much higher errors for the fit because the observed depolarization rate does not remain significantly below the fitted maximum of $f_{B}k_{\mathrm{BA}}\approx 0.85$ for [CrA‐ma]=8 μM. The differences between the two pulse shapes seem to be negligible for both hosts when the proper scaling factor between BP and dSNOB pulses is considered. The comparison of the theoretically achievable $\lambda_{\mathrm{depol}}$ confirms previous observations that CB6 is ca. 10^2^‐fold more efficient than CrA‐ma.^[10]^ Even for *P*_sat_ <25 mW BP equivalent, using 8 μM CrA‐ma is less efficient than using 2 μM CB6 despite the mentioned poor labelling efficiency for Xe exchanging in/out of the CB6 cavity. For medium and strong saturations (*P*_sat_ ≥50 mW BP equivalent), the faster exchange of Xe in the CB6 cavity reveals its high potential, resulting in much better depolarization, whereas the CrA‐ma does not show any improvement.

For saturation with the 100 mW block pulse/286 mW dSNOB pulse, monitoring of the temperature inside the 1 mL sample volume revealed that the temperature change did not exceed Δ_h_=0.3 °C for 50 repetitions of the pulse sequence with 5 s saturation when applying a train of 109 pulses with *t*_pulse_=45.76 ms (99.9 % duty cycle during *t*_sat_). This is expected because the total saturation time is almost identical to the 24 repetitions with *t*_sat_=10 s for the strongest saturation in Figure 5.

**Figure 11:**
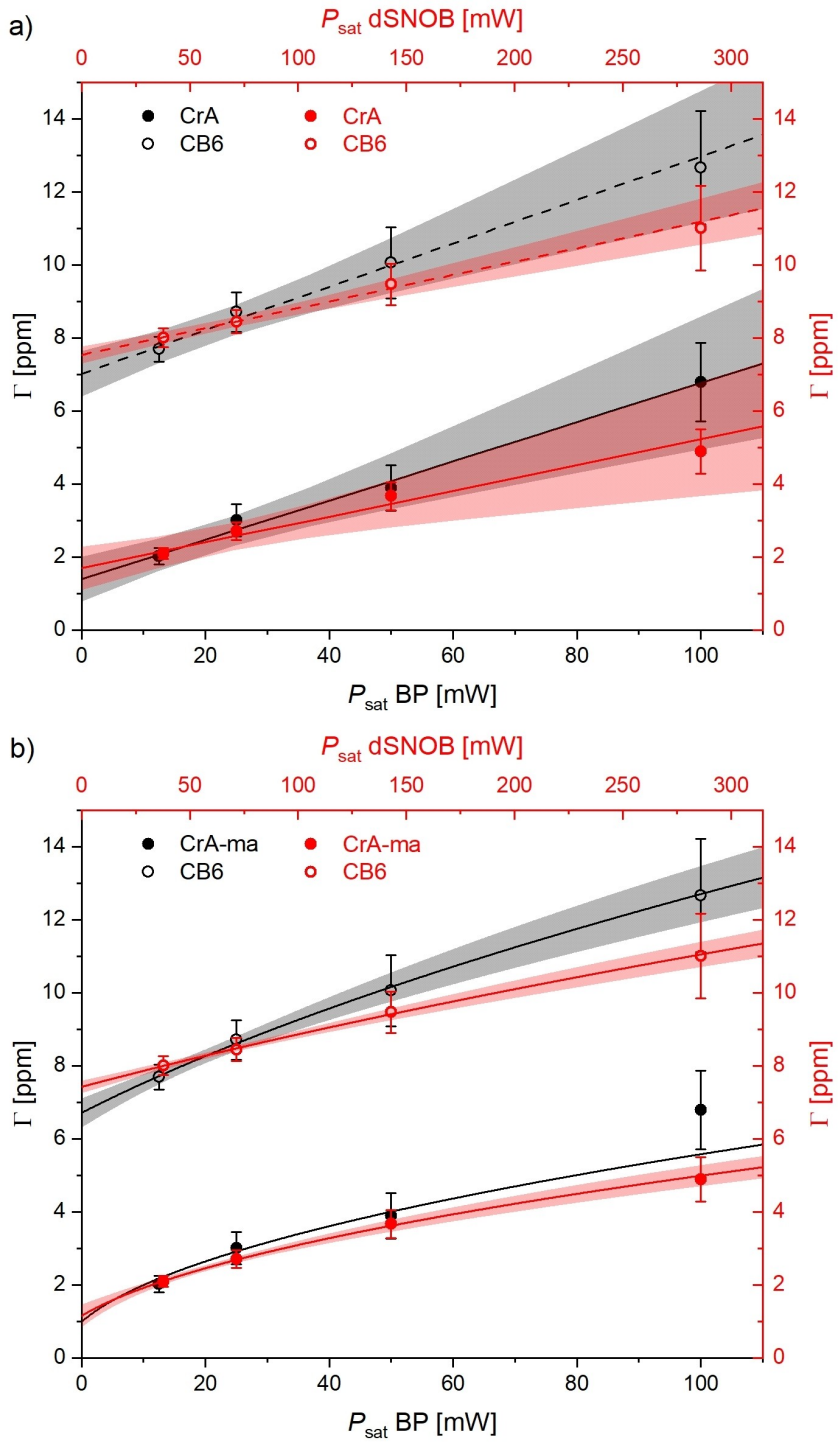
Line width analysis of CEST responses from z‐spectra in Figure 9 for block pulse (black) and dSNOB (red) saturation. (**a**) The approximated linear behavior is acceptable and illustrates that the line width is dominated by the contribution from the exchange rates $k_{\mathrm{BA}}$ . (**b**) Improved fitting results can be obtained from using the full equation $\Gamma =2k_{BA}\sqrt{s^{2}P_{\mathrm{sat}}+1}$ . The *y*‐intercepts allow estimating the exchange rate (1 ppm ≅ 696 s^−1^).

Line broadening effects in Figure 9 were also quantified and analyzed in Figure 11(a). $\Gamma \left(P_{\mathrm{sat}}\right)$ can indeed be modeled by a linear behavior, but the confidence suffers for the stronger saturation conditions. No significant differences can be recognized between the pulse shapes. The dSNOB pulse might cause slightly narrower responses, but the nominal bandwidth is irrelevant (see Suporting Information, Figure S7/8) compared to the contribution from $k_{BA}$ . A closer look at the *y*‐intercept for the CB6 data reveals that $k_{BA}=\Gamma \left(0\right)/2\approx 3.5\;\mathrm{ppm}\approx 2433\;s^{-1}$ . This is still larger than the strongest $\gamma B_{1}\approx 2294\;\;s^{-1}$ , but borderline for approximating the behavior as suggested. The scaling factor is ϵ≈0.94 and reaches even higher values for CrA‐ma with its slower exchange.

We also tested fitting the full equation $\Gamma =2k_{BA}\sqrt{\kappa^{2}P_{\mathrm{sat}}+1}$ to the obtained line widths (κ being again a scaling factor). The result is shown in Figure 11(b). The fit did not converge for the CrA‐ma saturation with a block pulse, but for the other three cases, the confidence could be improved. The deviation from linear behavior is not very pronounced, but the overall fit quality improves. The *y*‐intercepts allow estimating the exchange rates *k*_BA_: ~2460 s^−1^ for CB6 and ~402 s^−1^ for CrA‐ma under these solvent conditions.

Overall, the pulse parameters *P*_sat_ in Watt and $\gamma B_{1}$ in s^−1^ allow a comprehensive description of the RF effects regarding the characterization of the heating effects, depolarization efficiency, and line width in z‐spectra. The flip angle is a much less useful parameter because especially for Xe with its large chemical shift range, the effective field ${\vec{B}}_{\mathrm{eff}}$ in the rotating frame significantly tilts away from the equatorial plane, even for offsets that are rather small compared to the chemical shift range that needs to be covered. This makes the definition of a flip angle obsolete: applying *B*_1_=9 μT on a clinical scanner means that at an offset corresponding to 9 μT/3 T=3 ppm, the effective field is already 45° off the equatorial plane and the precessing magnetization will never tilt away more than 90° from the z‐axis. For the system used in this study, the numbers turn out quite similar. Stronger *B*_1_ is indeed achievable, but the static field and frequency offsets along the z‐spectrum also scale up. Our 100 mW pulse corresponds to *B*_1_=31 μT, and a remaining longitudinal field in the rotating frame would correspond to an offset of 31 μT/9.4 T=3.29 ppm. Saturation at any larger frequency offset does not flip the magnetization by more than 90° from the *z*‐axis.

### RF Heating for High‐Sensitivity CEST Detection

**Figure 12:**
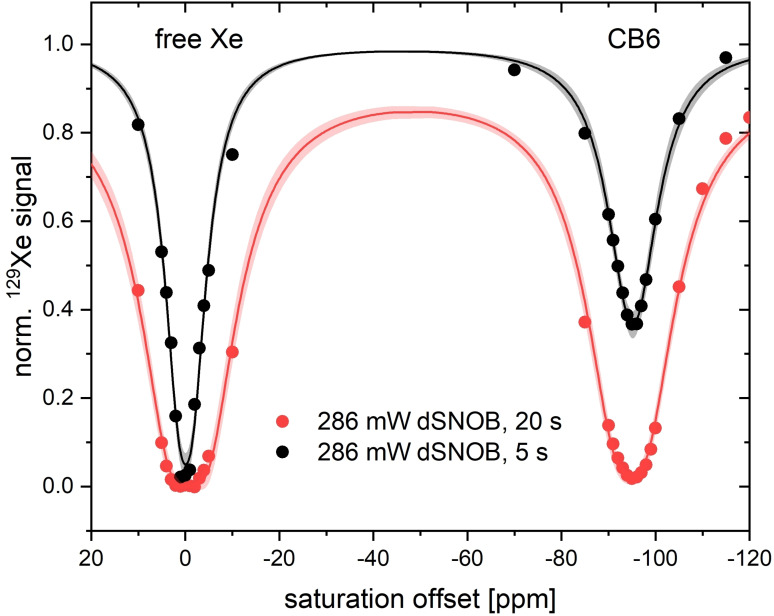
HyperCEST response of CB6 at 40 nM with 5 and 20 seconds high‐power saturation using a dSNOB pulse train. The baseline drops for the 20 s saturation according to *T*_1_ relaxation that occurs in the background. Data was fitted according to the FHC model with 95 % confidence level bands shown.

RF heating will be particularly relevant for detecting low concentrations of hosts with fast exchange kinetics where high saturation power and presumably long saturation times are required. We proceeded with investigating diluted samples of the hosts and focused on the CB6 signal while diluting the original concentrations by a factor of 10 and 50 (*i. e*., 0.2 and 0.04 μM for CB6; 0.8 and 0.16 μM for CrA‐ma). For the lowest concentration, a CEST effect with a short saturation time of 5 s was seen only for CB6. As expected, CB6 then becomes detectable at high power, but CrA‐ma remains undetectable upon such dilutions. Figure 12 shows z‐spectra for 40 nM CB6 with *t*_sat_=10 s and 20 s. Acquisition of these data sets led to an increase $\Delta_{h}\approx 0.4$ °C and 0.7 °C, respectively.

### Impact of Temperature Changes on Signal Intensity and Chemical Shift

The case in Figure 12 demonstrates the benefits of high power and long *t*_sat_, but the temperature changes of up to 0.7 °C occurring by the end of the acquisition time raise the question of further impacts on the z‐spectrum regarding a possibly slowly drifting resonance and a slowly increasing exchange rate. The CEST signal of CrA‐ma‐bound Xe exhibits changes in chemical shift and intensity due to a combined effect from an altered exchange rate and affinity constant.[^4^, ^22^] The CEST signal of CB6 has not yet been investigated in this regard. While the limited amplitude of RF‐induced heating (<1 °C) will cause only minor changes in the exchange rate, we decided to analyze z‐spectra over a wider temperature range for changes in chemical shifts and in *k*_BA_ (as reflected by changes in labelling efficiency and observed line width).

**Figure 13:**
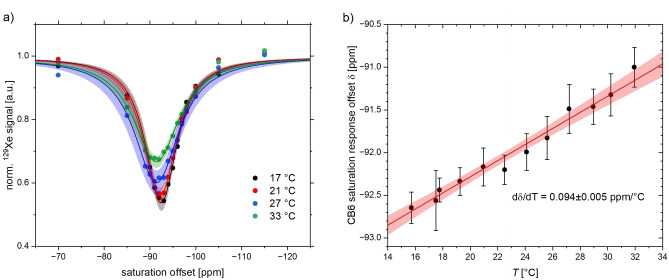
(**a**) Examples of HyperCEST spectra for CB6 (10 μM) for different VTU set points. A loss in CEST amplitude can be seen when the accelerated exchange rate is investigated with constant saturation of *P*_sat_=5 mW for a dSNOB pulse train with *t*_sat_=1 s (subset of 36 saturation offsets; exemplary 95 % confidence level bands are shown). (**b**) Linear fit with 95 % confidence level bands for the obtained line positions with error bars from using the FHC line shape model.

The changes in direct detection of bound Xe are shown for CrA‐ma in the Suporting Information, Figure S3. They are consistent with the known effects for this signal with decreasing intensity, increasing linewidth, and linear shift towards the solution signal upon increasing *T*. For the CB6 signal, the same idea was considered, but its limited solubility in combination with strongly pronounced line broadening compared to CrA‐ma makes the signal practically undetectable in conventional spectra. We therefore used CEST experiments under different VTU settings ranging from 17 °C to 37 °C for CB6 detection at a host concentration of 10 μM. The conditions were chosen such that a low‐power saturation of 5 mW block pulse equivalent would not cause additional heating, and we can work with the VTU‐controlled temperature. Figure 13 illustrates that the chemical shift of CB6‐bound Xe shows a detectable but small linear drift with ~0.094±0.005 ppm/°C. If the accelerated exchange is not accompanied by higher *P*_sat_ for increasing *T*, the CEST effect decreases upon heating the sample.

**Figure 14:**
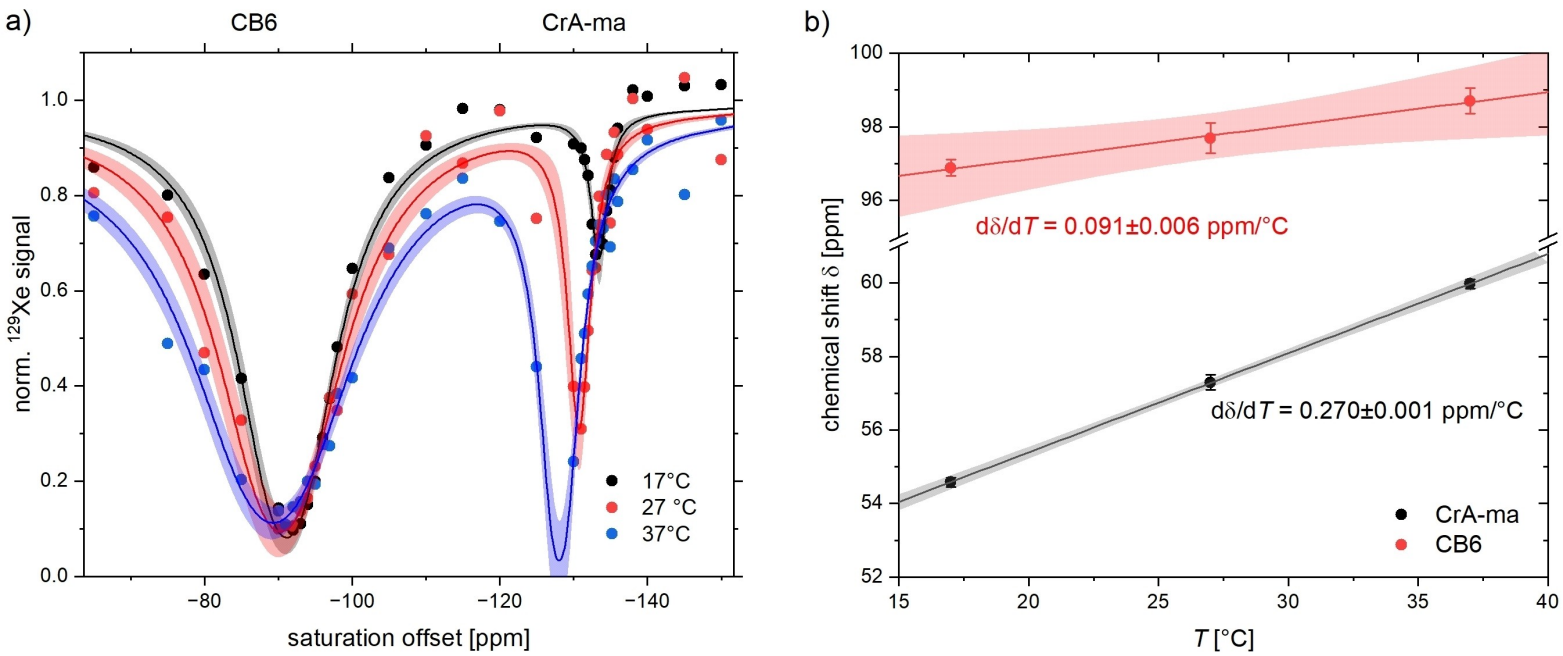
(**a**) HyperCEST spectra for CB6 (2 μM) in combination with CrA‐ma (8 μM) for different VTU set points. The CB6 signal shows little changes near full saturation whereas the CrA‐ma signal shifts and increases due to more efficient exchange upon heating; *P*_sat_=71.5 mW, dSNOB pulse train with *t*_sat_=5 s (subset of 52 saturation offsets). Data fitted with the FHC model and 95 % confidence level bands shown. **(b)** Linear fit for the obtained line positions in (a) with 95 % confidence level bands. Values for on‐resonant saturation offsets in (a) were converted into the chemical shift relative to the gas phase signal.

In order to compare these results to the temperature‐induced shift for the CrA‐ma signal, we decided to use the same method. Another CEST experiment with 2 μM CB6 and 8 μM CrA‐ma was set up and performed with a dSNOB pulse train with *P*_sat_=71.5 mW and *t*_sat_=5 s. These values were chosen to obtain a sufficiently strong CrA‐ma signal but avoid excessive saturation transfer from the previously used 10 μM CB6 sample. Figure 14 illustrates that the CrA‐ma signal benefits from the accelerated exchange upon heating (faster exchange), whereas the CB6 signal mainly broadens in the regime of almost full saturation throughout the temperature range. The obtained shifts are ~0.270±0.001 ppm/°C for CrA‐ma and 0.091±0.006 ppm/°C for CB6. Both are very consistent with previously reported values. This comparison from the same method illustrates that the CrA‐ma signal shifts ~3‐fold more sensitive with temperature under these solvent conditions. The approach of determining the temperature‐induced shifts from z‐spectra does not require very accurate phasing, as it is the case for directly observed signals that shift in the sub‐ppm range. Despite the relatively large line width in z‐spectra, the center position can be determined with high accuracy, and this is a big advantage for small effects like in the case of CB6. However, the absolute changes in Larmor frequency from unwanted RF heating that would be in the range of 1 °C are negligible for the typically acquired CEST responses.

## Conclusions

The balance between desired strong CEST responses and unwanted side effects from RF irradiation like heating and line broadening are particularly relevant for systems that allow access to relatively fast exchanging spins, *e. g*., HyperCEST or ParaCEST agents. Different saturation schemes and host systems have been investigated in this study with HyperCEST because ^129^Xe also offers the valuable feature of being an NMR thermometry sensor. Its sensing abilities require, however, careful elimination of the magnet drift when small temperature changes such as those from RF pulses are investigated.

Our results illustrate that the most relevant parameters are the total saturation time and the applied average RF power *P*_sat_. Pulse shape and particularly the number of pulses within the train are practically irrelevant for short inter‐pulse delays. We could not observe clear advantages of the previously suggested dSNOB pulse for more efficient CEST agents within the range of typically applied RF saturation. The relevant parameter for on‐resonant saturation in the case of Xe with well‐separated signals is the average *P*_sat_ or *B*_1_, whereas the pulse shape is not really relevant when comparing the pulses under fair conditions with the respective scaling factors. This clarifies misleading interpretations from previous work that claimed a sinc‐shaped pulse would be better than the block pulse.^[16]^. The pulse shape only seems to make a limited difference for off‐resonant effects, where the dSNOB pulse for high power with a defined bandwidth of 25 Hz appears to be slightly more selective than the cw saturation. This could be relevant for the response from CrA‐ma that shows signs of additional line broadening even for medium powers. However, this might only apply to z‐spectra with rather low noise level where the line shape can be reconstructed in a very accurate way to notice such differences.

For values of *P*_sat_ not exceeding 100 mW and $\sum_{\mathrm{loops}}t_{\mathrm{sat}}\leq \;$ 250 s of saturation over the entire experiment with all saturation offsets, temperature changes remained within ca. 0.3 °C. We found that in CEST experiments using different pulse shapes with comparative saturation power, RF deposition does not induce heating that causes relevant concomitant changes in the position of the CEST response or modifications of exchange rates. Such side effects of heating do not play a determinant role in many experimental designs. Even though the observed heating effects for *P*_sat_≤100 mW are small over the course of an experiment, it is important to realize that the consequences of RF‐induced heating are obviously not limited to clinical MRI systems with high absolute pulse powers. ParaCEST agents often show fast exchange kinetics that require *B*_1_~100 μT^[36]^ and this can be a few W for small RF resonators at high *B*_0_. For reference, it should be mentioned that *B*_1_=10 μT corresponds to *P*_sat_~10 mW of cw saturation for the 10 mm RF coil used in this study at 110.7 MHz (Larmor frequency for ^129^Xe at *B*_0_=9.4 T). *B*_1_=100 μT at this Larmor frequency for such coil geometry would then already require *P*_sat_=1 W. Switching to ^1^H CEST would increase this number even further due to the higher Larmor frequency. More extreme saturation conditions and especially longer total acquisition times may therefore require a revised approach.

*B*_1_ and *P*_sat_ are the suitable parameters to describe four key aspects for CEST experiments that are particularly relevant for promising fast‐exchanging hosts such as CB6. These aspects include:

– the applied power translates into a temperature change $\Delta_{\max}\propto P_{\mathrm{sat}}$ ;
– the labelling efficiency and therfore depolarization rate increases with $\lambda_{\mathrm{depol}}\propto \sqrt{P_{\mathrm{sat}}}$ to first approximation;
– the line width is dominated by the exchange rate *k*_BA,_ but the additional term increases with $\lambda_{\mathrm{depol}}\propto P_{\mathrm{sat}}$ to first approximation;
– the estimation of off‐resonant effects is obtained by comparing $\gamma B_{1}$ to the offset from the Larmor frequency of the saturated pool, $\Delta \omega_{B}$ .

This complex interplay of several parameters should be considered when optimizing the signal amplification that comes from CEST agents with desirable large chemical shift separations.

## Supporting Information Summary

The authors provide additional data within the Supporting Information.

## Conflict of Interests

The authors declare no conflict of interest.

1

## Supporting information

As a service to our authors and readers, this journal provides supporting information supplied by the authors. Such materials are peer reviewed and may be re‐organized for online delivery, but are not copy‐edited or typeset. Technical support issues arising from supporting information (other than missing files) should be addressed to the authors.

Supporting Information

## Data Availability

The data that support the findings of this study are available from the corresponding author upon reasonable request.
