# Supplementary material for: RF Heating Effects in CEST NMR with Hyperpolarized 129Xe Considering Different Spin Exchange Kinetics and Saturation Schemes
Source: Chemphyschem. 2025 Feb 5;26(8):e202401037. doi: 10.1002/cphc.202401037 (PMC12005132; doi:10.1002/cphc.202401037)
Supplement: Supplementary file 1 — Supporting Information [file CPHC-26-e202401037-s001.pdf]

ChemPhysChem

Supporting Information

RF Heating Effects in CEST NMR with Hyperpolarized ^{129}Xe Considering Different Spin Exchange Kinetics and Saturation Schemes

David Hernandez-Solarte and Leif Schröder*

Supplementary Information

for

RF Heating Effects in CEST MRI with Hyperpolarized ^{129}Xe Considering Different Spin Exchange Kinetics and Saturation Schemes

David Hernandez[1,2], Leif Schröder[1-4]

[1]Translational Molecular Imaging (E280), Deutsches Krebsforschungszentrum (DKFZ), 69120 Heidelberg, Germany

[2]Graduate Research Center 2260 BIOphysical Quantitative Imaging Towards Clinical Diagnosis (BIOQIC), Department of Radiology, Charité - Universitätsmedizin Berlin, 10117 Berlin, Germany

[3]Department of Physics and Astronomy, Ruprecht-Karls University Heidelberg, 69120 Heidelberg, Germany

[4]German Consortium for Translational Cancer Research (DKTK), Core Site Heidelberg, 69120 Heidelberg, Germany

Table of Contents

1. Pulse sequences 2
2. Temperature-dependent CrA-ma chemical shift 3
3. ^{129}Xe chemical shift with decreasing temperature 3
4. Heating comparison between 400 MHz (^{1}H) and 110 MHz (^{129}Xe) 4
5. Heating effect using different shaped pulses 4
6. Pulse saturation with different bandwidth 5
7. Pulse conversion table 6

1. Pulse sequences

Two main sequences were used, one for the heating (thermometry) experiments, where it was iterated over the thre phases of an experiment16 initial scans with no power, followed by 24 with RF power activated to induce heating, and 88 scans at the end again with no power to let the system recover to ambient temperature. The second sequence worked in a similar setup with the possibility to change the saturation time as required but it was iterated over the saturation frequency offset list (z-spectroscopy).

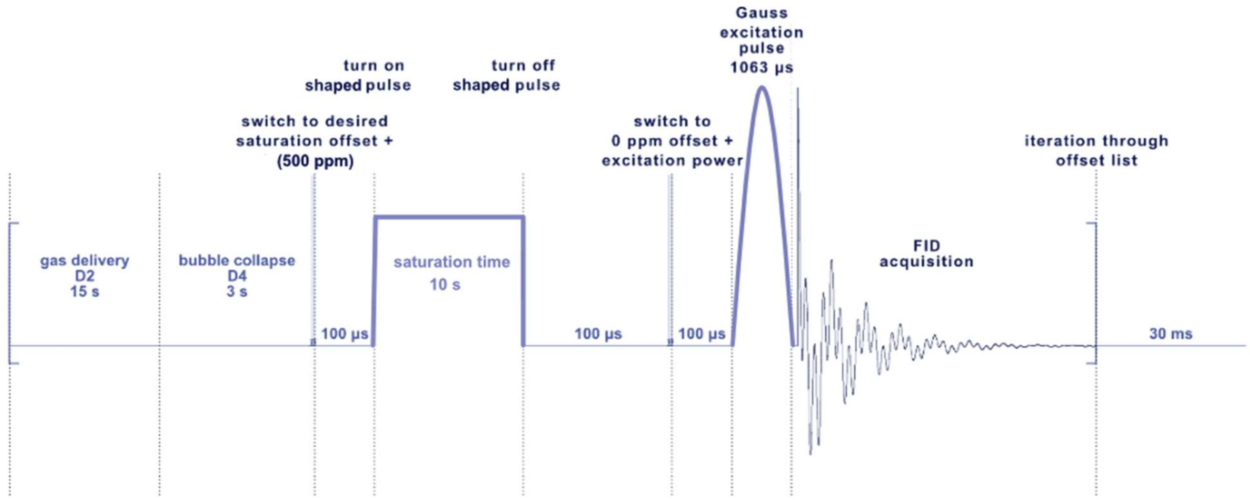

Figure S1: Thermometry sequence

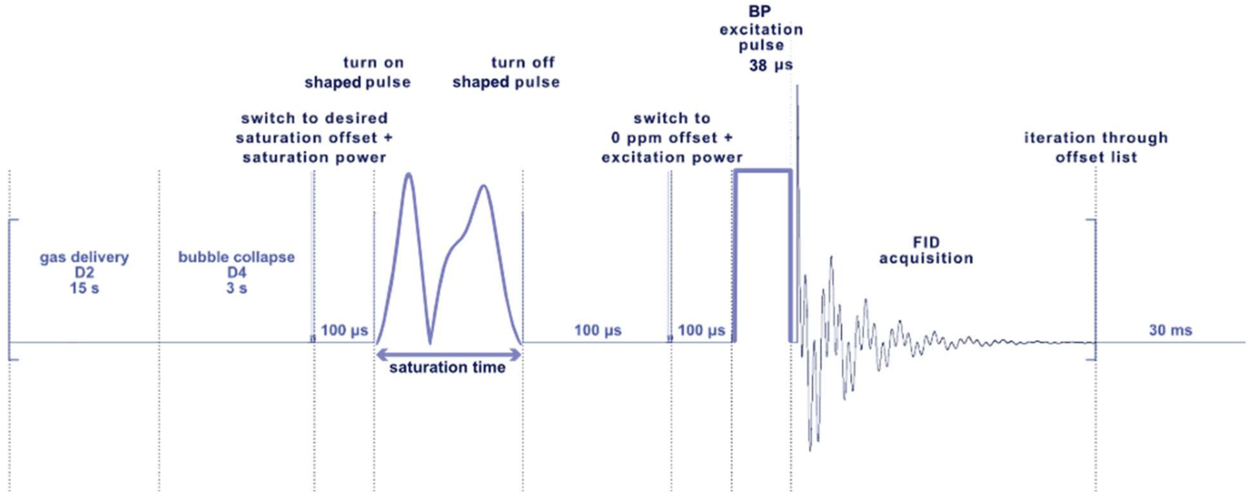

Figure S2: Z-spectroscopy sequence

2. Temperature-dependent CrA-ma chemical shift

The cryptophane-A monoacid chemical shift was evauated using direct spectra acquisition that shows increased signal with lower temperature (due to higher binding affinity); it also shows a shift up field with increasing temperature (gas signal referenced as 0 ppm).

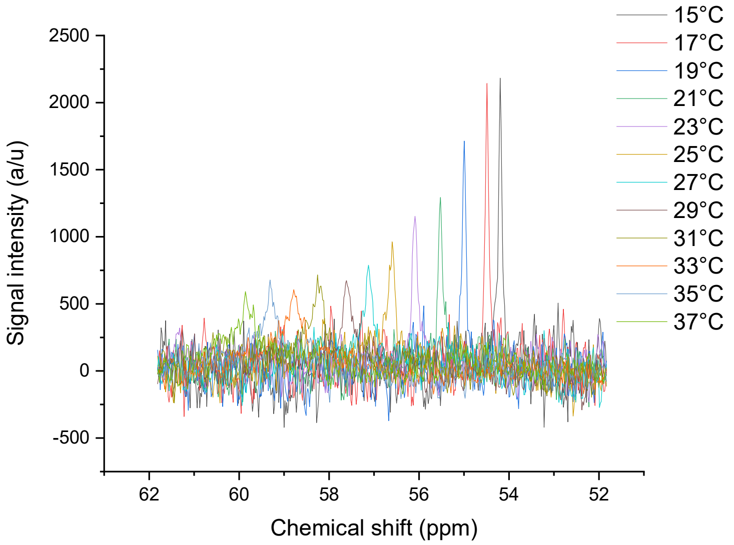

Figure S3: Direct CrA-ma spectroscopy illustrating temperature shifts

3. ^{129}Xe chemical shift with decreasing temperature

The response from the chemical shift using decreasing temperature steps shows that the parabolic behavior works with either heating up or cooling down the environment.

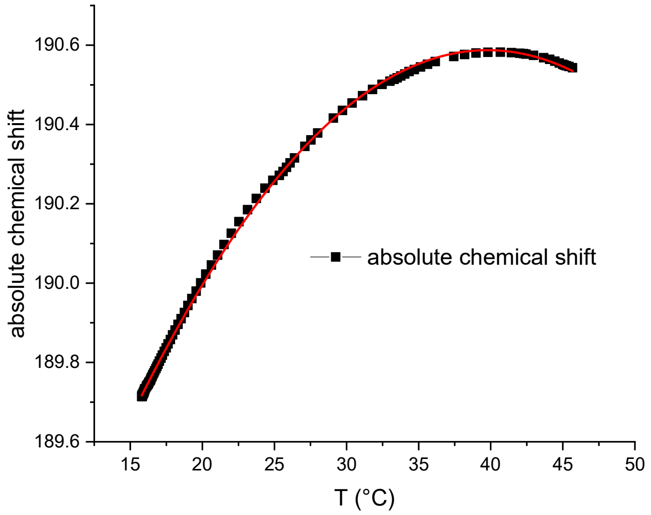

Figure S4: Chemical shift with decreasing temperature steps

4. Heating comparison between 400 MHz (^1H) and 110 MHz (^{129}Xe)

Comparison between both RF channels shows that the systems experience the same temperature regardless of the coil used to apply the saturation RF pulses.

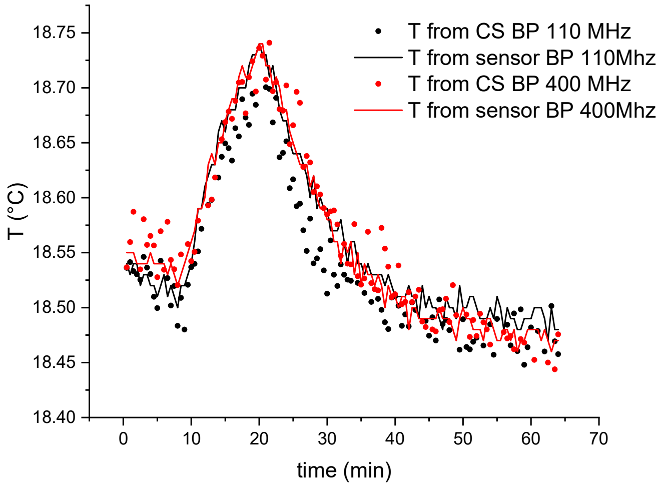

Figure S5: Temperature using 400MHz and 110MHz channels

5. Heating effect using different shaped pulses

The heating generated from pulses with equivalent power is the same, only t_{sat} is relevant.

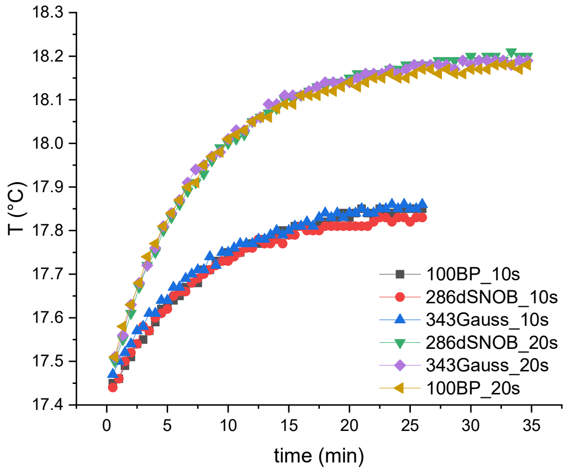

Figure S6: Temperature comparison using different pulses with different saturation times. The first number in the pulse name gives the peak power in mW according to the scaling factor given by topspin relative to a 100 mW block pulse.

6. Pulse saturation with different bandwidth

The performance of different shaped pulses with bandwidths of 25 Hz or 100 Hz was yields very similar results. The block pulse for reference yields a slightly deeper and wider CEEST response foor the faster exchanging Xe in CB6.

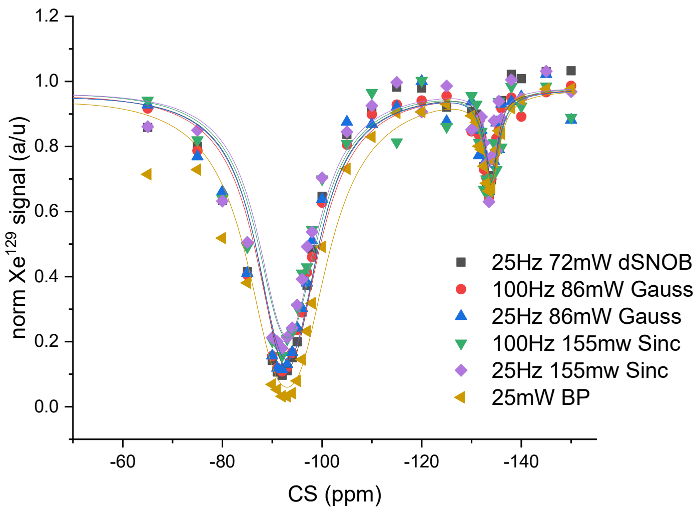

Figure S7: CEST spectra using different shaped pulses with 25Hz and 100Hz

For a 25 Hz dSNOB pulse, the duration is 45.76 ms; if this time is kept for other shaped pulses, their bandwidth is different. Even if the number of pulses during the entire RF saturation duration is different, the response is almost identical., The block pulse yields again a slightly stronger and wider response.

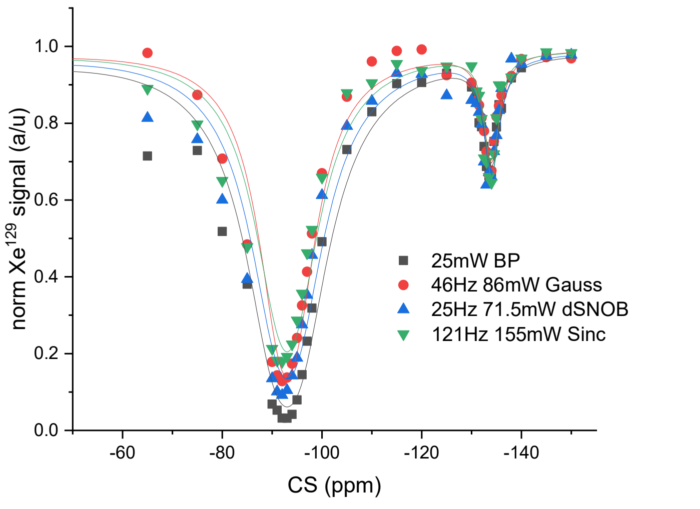

Figure S8: CEST using different shaped pulses with the duration of a 25Hz dSNOB with 25 mW block pulse equivalent power.

7. Pulse conversion table

This table gives the equivalent power for selected pulses, compared to a block pulse to generate the same heating effect, and a table with the conversion from mW to µT to have the same B_1 strength area under the curve.

Table S1: Equivalent pulse power for different shapes

Pulse shape	Block Pulse	dSNOB	Gauss	Sinc
Power (mW)	100	286	343	621
	80	228.8	274.4	496.8
	60	171.6	205.8	372.6
	50	143	171.5	310.5
	40	114.4	137.2	248.4
	25	71.5	85.75	155.25
	12.5	35.75	42.875	77.625

Table S2: Power conversion from mW to µT

BP power (mW)	Block Pulse (µT)	dSNOB (µT)	Gauss (µT)	Sinc (µT)
100	31.054	52.518	57.51	77.388
80	27.776	46.974	51.44	69.218
60	24.055	40.680	44.55	59.944
50	21.959	37.136	40.67	54.721
40	19.640	33.215	36.38	48.944
25	15.527	26.259	28.76	38.694
20	13.888	23.487	25.72	34.609
